# Exploring the glucose-lowering and anti-inflammatory immune mechanism of artemether by AMPK/mTOR pathway and microbiome based on multi-omics

**DOI:** 10.3389/fphar.2025.1520439

**Published:** 2025-02-19

**Authors:** Tao Jiang, Peipei Du, Dongxia Liu, Hetao Chen, Yujin Ma, Bin Hu, Jingna Li, Hongwei Jiang, Xuejiao Li

**Affiliations:** ^1^ Department of Clinical Laboratory, The First Affiliated Hospital, and College of Clinical Medicine of Henan University of Science and Technology, Luoyang, China; ^2^ Luoyang Key Laboratory of Clinical Multiomics and Translational Medicine, Key Laboratory of Hereditary Rare Diseases of Health Commission of Henan Province, Henan Key Laboratory of Rare Diseases, Endocrinology and Metabolism Center, The First Affiliated Hospital, and College of Clinical Medicine of Henan University of Science and Technology, Luoyang, China; ^3^ Department of Pharmacy, The First Affiliated Hospital, and College of Clinical Medicine of Henan University of Science and Technology, Luoyang, China

**Keywords:** T2DM, network pharmacology, artemether, metabolomics, gut microbiota, AMPK/mTOR pathway

## Abstract

**Background:**

Diabetes mellitus (DM) is a metabolic disease with high morbidity, which significantly affects human life and health expenditures. Previous studies have demonstrated that artemether (ATM) has anti-diabetes and anti-inflammation activities, but its mechanism has not been fully elucidated. This research aimed to elucidate the impact of ATM on glucolipid metabolism in a type 2 diabetes mellitus (T2DM) model db/db mice and what kind of role the gut microbiota played, and explored the underlying mechanisms involved.

**Methods:**

C57BL/KsJ-db/db mice were treated with 80 and 160 mg/kg of ATM for 8 weeks, with metformin as a positive control.

**Results:**

ATM treatment (160 mg/kg) observably ameliorated insulin resistance (IR), hyperglycemia, hyperlipemia and pathological injury in the liver and pancreas. In addition, ATM significantly decreased the expression of TNF-α, IL-1β, IL-6, NF-κB and IL-17A, and significantly increased the level of IL-10 in diabetic mice. 16S rRNA sequencing and targeted GC-MS metabolomics result indicated that ATM restored gut microbiota dysbiosis based on increasing beneficial bacteria *Lactobacillus* and reducing pathogenic bacteria *Helicobacter* and *Prevotella* leading to the accumulation of propionic and valeric acids and the reduction of lipopolysaccharides (LPS) release, intestinal inflammation and intestinal barrier damage. Network pharmacology and metabolomics identified the AMPK/mTOR pathway as the main signaling involved in ATM improves glucolipid metabolism and inflammation in T2DM. Western blotting results revealed that ATM suppressed the phosphorylation of mTOR, P38, P65, IKBα and IRS1 whlie increased the levels of p-AMPK, TLR4, and occludin in mice liver and colon.

**Conclusion:**

Taken together, ATM may modulate the composition of gut microbiota, increasing the abundance of *Lactobacillus*, which in turn elevates the levels of SCFAs. The elevation of SCFAs, especially propionic acid, may activate the AMPK/mTOR pathway, leading to a decrease in the levels of TNF-α, IL-1β, IL-6, NF-κB, and IL-17A, while increasing the levels of IL-10, thereby alleviating the inflammatory state and improving glucolipid metabolic disorder in T2DM. These findings laid a theoretical foundation for the clinical application of ATM in T2DM.

## 1 Introduction

DM is a complex chronic metabolic-related disease with the features of insulin dysfunction, hyperglycemia, metabolic disorders, and chronic low-grade inflammation. DM is responsible for over one million deaths annually on a global scale (Khan et al., 2020), and it is projected that the number of individuals affected by diabetes will escalate to 783 million by 2045 (Sun et al., 2022). Among them, the key factor of T2DM are IR and relative insufficiency of insulin secretion, which is mainly manifested as IR in organs, thus causing chronic low-grade inflammation of the whole body (Johnson and Luciani, 2010; Chen et al., 2024). T2DM is frequently associated with metabolic dysfunctions, such as obesity, fatty liver disease, and dyslipidemia, which typically precede the onset of T2DM and are regarded as upstream conditions. IR and islet *β*-cell dysfunction are induced by dyslipidemia, hyperglycemia, and other metabolic disorders via several shared pathways, including inflammation, endoplasmic reticulum stress, oxidative stress, and ectopic lipid accumulation (Lu et al., 2024). Subsequently, this can lead to pancreatic *β*-cell dysfunction, and cause a range of serious complications such as renal failure, heart attacks, and vision loss (Saedi et al., 2016; Jia et al., 2018; Chen et al., 2024). Although sulfonylureas, thiazolidinediones, incretin and other drugs have shown excellent clinical efficacy for the treatment of diabetes, they also cause toxic and side effects, such as hypoglycemia, abdominal pain, nausea, vomiting, organ function damage, etc (McGovern et al., 2018; Tawulie et al., 2023). Consequently, there is a compelling necessity to develop novel therapeutic agents that can effectively and specifically address the treatment of T2DM.

Compared with traditional hypoglycemic drugs, Chinese herbal medicine contains various natural ingredients, exhibits the features of multi-targets and good efficacy, and has attracted wide concern in the therapy of T2DM. Artemisinin, a novel lactone compound obtained from *Artemisia annua* Linn (Asteraceae), is mainly used to treat malaria. It exhibits anti-inflammatory and immunomodulatory activities and has unique advantages in treating metabolic diseases, such as DM, and obesity (Lu et al., 2016; Jiang et al., 2020). They improve DM and its corresponding complications by ameliorating IR, reducing inflammation, defending islet *β* cells, or even inducing islet *α* cells to transform into *β*-like cells (Li et al., 2017; Chen et al., 2024). In recent study, we found that artemisinin derivative ATM can significantly improve blood glucose metabolism and IR in db/db mice, prevent obesity and alleviate fatty liver (Guo et al., 2018). Moreover, ATM may upregulate GLUT-4 and IR*β* protein expression via the AMPK pathway, thereby improving the hyperlipemia and IR of diabetes mice (Fu et al., 2020). These studies indicated that ATM exerts a beneficial effect in the treatment of T2DM. However, the molecular mechanisms underlying ATM’s therapeutic effects in T2DM still needs to be fully elucidated.

The gut microbiota is a vital factor in disease by shaping immune response, and metabolism (Yang et al., 2023). Numerous studies found that changes in the gut microbiome are the drivers of the metabolic inflammation observed in T2DM. Changes in the gut microbiota can affect the host’s glucose metabolism (Scheithauer et al., 2020). Hyperglycemia will increase intestinal permeability and destroy the intestinal mucosal barrier, and the dysfunction of the intestinal barrier will promote the absorption of pathogens and toxins, thus triggering the abnormal immune system (Yao et al., 2017; Thaiss et al., 2018). An abnormal intestinal immune system can induce a chronic inflammatory response, which will further aggravate IR and hyperglycemia conversely (Weaver et al., 2015; Genda et al., 2017). Recent research revealed that ATM can significantly improve gut microbiota imbalance in high-fat and fructose diet mice by increasing ZO-1 and occludin levels, decreasing TNF-α and IL-1β expression, and alleviating intestinal barrier dysfunction and inflammatory response (Ren et al., 2023). Consequently, this study employed a microbiome analysis to investigate the role of the intestinal flora in the amelioration of T2DM through the administration of ATM.

In this study, we utilized C57BL/KsJ-db/db mice as a DM model to conduct a comprehensive pharmacodynamic experiment about ATM improving glucose and lipid metabolism and inflammation in T2DM. Subsequently, a multi-omics research strategy consisting of the gut microbiome, metabolomics, and network pharmacology was applied to systematically uncover the hypoglycemic and lipid-reducing pathways, and identify the potential targets and scientific mechanisms of ATM in ameliorating T2DM and related metabolic disorders. The present study provides new insights into the mechanism of ATM in lowering blood glucose and lipids. It will lay a scientific foundation for the clinical application of *Artemisia* herbal medicines for reducing blood glucose and inflammation, improving metabolic disorders, and preventing and treating T2DM.

## 2 Materials and methods

### 2.1 Chemicals and reagents

Artemether (ATM, S31395-5G) and metformin (MET, S30880-25G) with HPLC purity ≥98% were bought from Yuanye Biotechnology Co., Ltd. (Shanghai, China). 1.25% Tribromoethanol (2071A-8-15) was bought from Aibei Biotechnology Co., Ltd. (Nanjing, China). Methylcellulose (m8070) was obtained from Suolaibao Biotechnology Co., Ltd. (Beijing, China). Acetonitrile, methanol and formic acid (MS grade) were provided by Thermo Fisher Scientific Inc. (Massachusetts, UAS). RIPA lysis buffer (P0013B), BCA protein assay kit (P0012), and SDS-PAGE protein sample loading buffer (P0015L) were obtained from Beyotime Biotechnology Co., Ltd. (Shanghai, China). TLR4 (AF7017), p-P65 (AF 2006), P65 (AF5006), p-P38 (AF3456), P38 (AF6455), p-mTOR (AF3310), mTOR (AF6308), Occludin (AF7504), IKB*α* (AF5002), claudin-1 (AF6919), claudin-1 (AF6919), ZO-1 (AF5145), p-AMPK (AF6423), AMPK (AF3423) antibodies were purchased from Affinity Biosciences LTD. (Jiangsu, China). GAPDH (M9002) and anti-Rabit IgG (S2001) were obtained from Simu Biotechnology (Tianjin, China). Anti-Mouse IgG (AS003) was provided by ABclonal Technology (Wuhan, China).

### 2.2 Animal experiment

48 Male C57BL/KsJ-db/db mice and 12 C57BL/KsJ-db/m mice (6 weeks old) were bought from Beijing Vital River Laboratory Animal Technology Co., LTD. (Beijing, China). All mice were kept in an environment with a temperature of 23°C ± 2°C and relative humidity of 50% ± 10%. The mice were acclimated for 2 weeks before the experiment began under standard conditions of 12-h light and no constraint in food and water. The animal studies were approved by the First Affiliated Hospital of Henan University of Science and Technology (Luoyang, China, NO. 2023–005), and carried out by the principle of 3Rs.

12 male C57BL/KsJ-db/m mice were selected as the normal control group (NC), and 48 male C57BL/KsJ-db/db mice were divided into four groups (12 mice per group): model group (MD), low-dose artemether group (ATM-L), high-dose artemether group (ATM-H) and MET positive control group. The dose for the ATM-L group was 80 mg/kg, the dose for the ATM-H group was 160 mg/kg, the dose for the MET group was 300 mg/kg, and the drug intervention period was 8 weeks. The ATM and MET were administered via gavage daily at a volume of 0.2 mL per 40 g body weight, dissolved in a 1% methylcellulose solution. The NC group received a 0.2 mL/40 g saline solution via gavage.

Each group of mice was equally housed in three separate cages with no constraint in water and food. The 24-h average food intake, average water intake, fasting blood glucose (FBG) and body weight were detected every 3 days. The mice were fasted for 12 h before collecting blood samples to measure FBG levels based on Accu-Chek device (Roche, Switzerland). Random blood glucose was monitored every 2 weeks. After 8 weeks, 1.25% tribromoethanol (0.02 mL/g) was injected intraperitoneally to anesthetize mice (Cho et al., 2011), and blood samples were collected to detect biochemical indicators, and inflammatory factors, and perform IPGTT and IPITT experiments according to the previous method (Guo et al., 2018). To exclude the influence of stress state on the experimental results, the IPGTT and IPITT experiments were performed at least 48 h apart. The livers and pancreas of the mice were dissected for pathological experiments.

### 2.3 Biochemical measurements

Biochemical indicators of ALT, TC were detected using a biochemical analyzer (Mindray BS-240VET, China) and ELISA kits (Ruixin Biotech, China) for fasting insulin (FINS, Cat.NO.OM21914), c-peptide (C-P, Cat.NO.OM20052), glycated heamoglobin A1c (GHbA1c, Cat.NO.OM20652), TNF-α (RX202412M), IL-1β (RX203063M), IL-6 (RX203049M), NF-κB (RX202896M), IL-17A (RX203066M), IL-10 (RX203075M), IL-22 (RX203059M), TGF-*β*1 (RX202402M) and lipopolysaccharide (LPS, RXJ202425M).

### 2.4 H&E staining

After liver, pancreas and colon collections, some tissues were embedded in paraffin after 4% formalin immersion. After slicing and dewaxing, the slices were stained using H&E solution (G1076, Servicebio, Wuhan, China). The slices were sealed using neutral gum. Finally, an electron microscope was applied to collect and analyze the alterations of these tissues.

### 2.5 Immunohistochemistry

The sections were dewaxed by convention. Microwave antigen retrieval was conducted, subsequently blocked with goat serum at 37°C for 1 h. Primary antibodies caspase-3 (1:500, GB11532), IL-1β (1:500, GB11113), IL-6 (1:200, GB11117), NF-κB (1:1000, GB11997), TLR4 (1:1000, GB11519), TNF-α (1:500, GB11188) (Servicebio, Wuhan, China) were added dropwise and incubated overnight. After PBS washing, the secondary antibodies were incubated, followed by hematoxylin counterstaining. Then, the sections were sealed with neutral after dehydration. The positive expression area with brown-yellow particles was interpreted by randomly selecting four fields of view in each group under a microscope (E100, Nikon, Japan).

### 2.6 Network pharmacology

The PharmMapper database, STITCH and SwissTargetsPrediction were used to retrieve the drug targets of ATM respectively. A search for “T2DM”, “inflammation” and “immunity” were performed in GeneCards, OMIM, and DisGeNET databases to identify the corresponding targets. The intersection targets were imported into the STRING database for PPI analysis. GO and KEGG pathway enrichment analysis of intersection targets performed using the Metascape database. Finally, the biological network of the “ATM active ingredient-key target-signaling pathway” was visualized via Cytoscape 3.7.0 software.

### 2.7 Fecal 16S rRNA sequencing and SCFAs analysis

For colon microbiota analysis, total microbial genomic DNA was extracted, and 16S rRNA genes were sequenced in the Illumina platform of Baipu Biotechnology Co., LTD. (Shanghai, China). Bacteria with a length of about 480bp were selected to amplify the hypervariable region V3-V4 of the 16S rRNA gene by PCR with primer pairs. PCR products were quantified using a Microplate reader (BioTek, FLx800). The TruSeq Nano DNA LT Library Prep Kit was applied for library construction. Eligible libraries were sequenced on both ends on an Illumina NovaSeq machine. The other details are the same as previously described (Sun et al., 2024).

For the targeted analysis of feces SCFAs, GC-MS analysis was utilized. In this experiment, 50 mg of feces and 0.5 mL of Mili Q water were homogenized on an HF-24 homogenizer (Hefan, Shanghai, China). The standard SCFAs mixture included propionic acid, acetic acid, isobutyric acid, butyric acid, isovaleric acid and valeric acid (Sigma-Aldrich, Shanghai, China). The other detailed process refers to the former research (Zhang S. et al., 2019).

### 2.8 Metabolomic analysis

After collection, each hepatic tissue sample (50 mg) was stored in a microcentrifuge tube, homogenized for 3 min, and metabolites were extracted with 1 mL of a precooled methanol/water (4:1), with high speed at 4°C. The supernatant was put into another tube, vaporized, and redissolved with 100 μL of methanol/water (4:1). Non-targeted metabolite analysis was performed using Waters UHPLC-MS/MS system (Waters, Milford, MA, United States) with a UHPLC C18 column (2.1 × 100 mm, 1.7 µm, Waters, United States) at 40°C by an elution gradient of water with 0.01% formic acid (A) and acetonitrile (B) with a rate of 300 μL over 15 min: 0–2 min (0% B), 2–6 min (0%–48% B), 6–10 min (48%–100% B), 10–12 min (100% B), 12–12.1 min (100%–0% B), 12.1–15 min (0% B). MS data was operated at two modes of electrospray ionization (ESI). The obtained raw data files were treated based on MS-DIAL for peak integration, calibration, and quantification of all metabolites. R, version 4.0.3, and the R package were used for all multivariate data analyses and modeling.

### 2.9 Western blotting (WB)

Total proteins were extracted from the liver and colon tissue homogenate with RIPA buffer and quantified using a BCA detection kit (Biotime, Shanghai, China). Then the samples were separated and transferred to the PVDF membrane. After blocking of non-specific binding sites for 2 h, the membrane was probed for 12 h at 4°C with primary antibodies TLR4, p-AMPK, AMPK, p-P65, P65, p-P38, P38, p-mTOR, mTOR, ZO-1, IKB*α*, p-IKB*α*, claudin-1, occludin (Affinity, Jiangsu, China) and mouse polyclonal GAPDH (Simu Biology, Tianjin, China). The bands were visible by enhanced chemiluminescence using a chemiluminescence apparatus (JUNYI, MINICHEMI) after incubation with the secondary antibody (ABclonal, Wuhan, China) for 2 h. ImageJ 1.8.0 software was applied for densitometric analysis of protein levels and the values were calibrated to those of GAPDH.

### 2.10 Molecular docking

Molecular docking experiments are a computational chemistry technique used to predict the binding mode and affinity between small molecule ligands and biological macromolecular receptors. Initially, the receptor structure is retrieved from the Protein Data Bank (PDB), where impurities are removed and hydrogen atoms are added. The ligand structure is either sourced from chemical databases or designed *de novo*, followed by the generation of a three-dimensional structure and energy minimization. Subsequently, the active site of the receptor is identified, typically based on known ligand-binding sites or homology modeling. Multiple conformations of the ligand are then generated, and molecular docking software such as AutoDock is employed to search for the optimal binding pose within the active site. Upon completion of the calculations, the results are analyzed using visualization tools to assess binding energy and molecular interactions.

### 2.11 Statistical analysis

GraphPad 10.1.2 software was applied to statistical analysis. Data were expressed as the mean ± SD of at least three independent experiments. For normally distributed data, one-way ANOVA was employed to compare multiple groups of samples. Additionally, t-tests were utilized for comparisons involving two groups in metabolomics. For abnormally distributed data, the variance was tested by a rank sum test. *P* < 0.05 means that the difference is statistically significant.

## 3 Result

### 3.1 ATM ameliorates the glucose and lipid metabolism disorders in db/db mice

**FIGURE 1:**
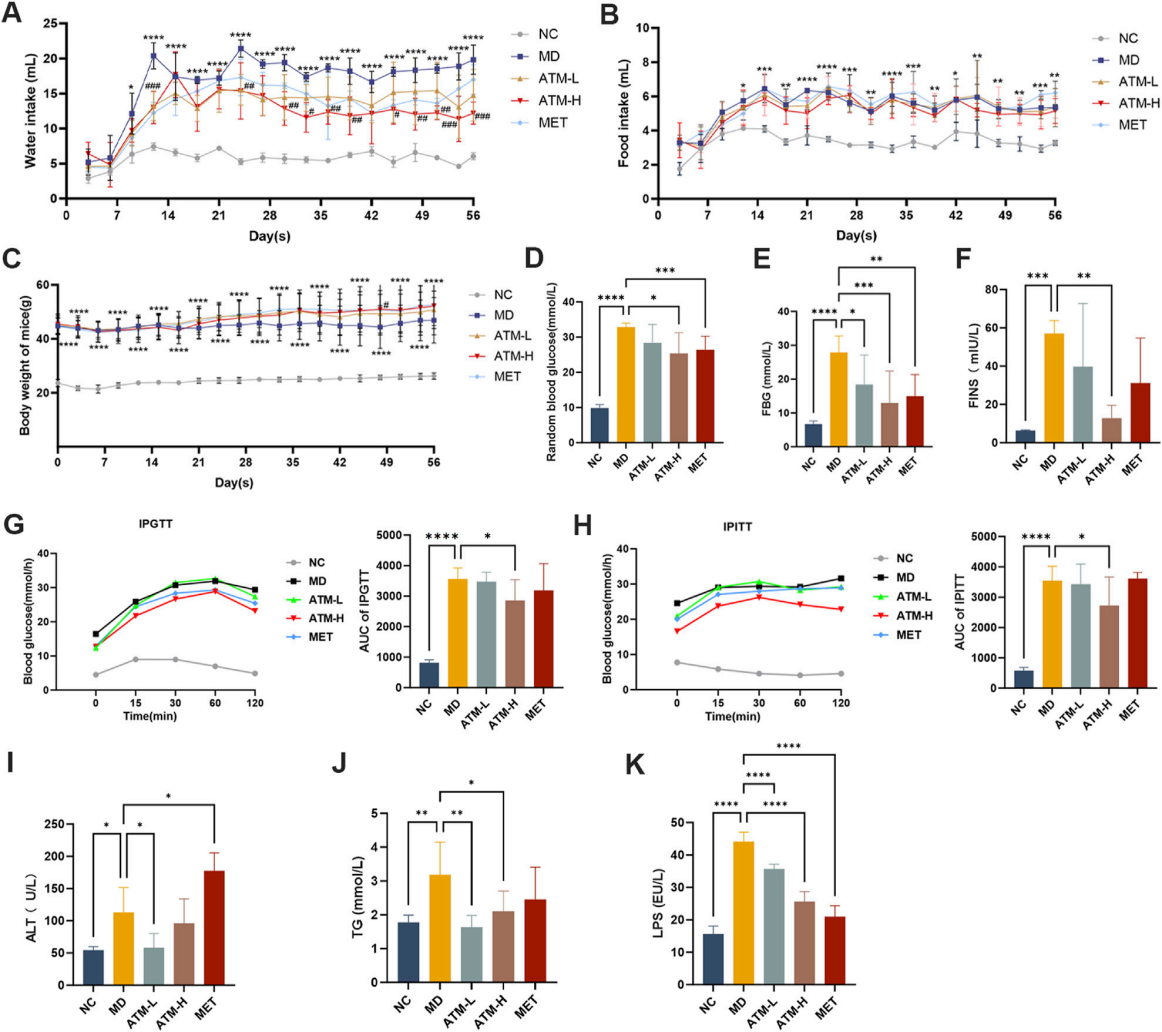
Evaluation of ATM Drug Efficacy. **(A–C)** Changes in the mice’s water intake, diet and body weight were measured every 3 days during ATM intervention. **(D–F)** Levels of random blood glucose, FBG, and FINS of mice. **(G, H)** Results of blood glucose and AUC of IPGTT test and IPITT test. **(I–K)** ALT, TG, and LPS levels in each group. *P < 0.05, **P < 0.01, ***P < 0.001, ****P < 0.0001 VS the NC group; ^#^ *P* < 0.05, ^##^ *P* < 0.01, ^###^ *P* < 0.001, ^####^ *P* < 0.0001 VS the MD group.

Compared to the NC group, the food, water intake and body weight of the MD group were obviously enhanced (Figures 1A–C, *P* < 0.05). The water intake of the ATM-H group was significantly decreased from day 12 than that of the MD group (Figure 1A, *P* < 0.001). The food intake of all ATM treatment groups was slightly decreased, while body weight was slightly increased (Figures 1B, C). Moreover, the levels of random blood glucose, FBG, and FINS in the MD group were significantly higher compared to the NC group (Figures 1D–F, *P* < 0.001), and the levels of random blood glucose, FBG, and FINS in the ATM-H group were obviously lower compared to MD group (Figures 1D–F, *P* < 0.05). FBG levels were obviously reduced in the ATM-L group (Figure 1E, *P* < 0.05). IPGTT test results showed that the blood glucose of every group reached the peak at 60 min, while the ATM-H group showed lower blood glucose levels (Figure 1G). The AUC of the MD group was more observably increased than the NC group (Figure 1G, *P* < 0.0001). The AUC of the ATM-H group was significantly reduced than the model group (Figure 1G, *P* < 0.05). And the blood glucose of the ATM-H group decreased substantially at each time point with AUC significantly lower than the MD group (Figure 1H, *P* < 0.05) in the IPITT test. The above results displayed that ATM has a significant hypoglycemic effect. Moreover, the serum TG of the MD group was observably increased (Figure 1J, *P* < 0.05) than the NC group, and was significantly reversed by ATM treatment (Figure 1J, ATM-L, *P* < 0.01; ATM-H, *P* < 0.05). The above result suggested that ATM intervention could, to a certain extent, alleviate the dyslipidemia in T2DM.

### 3.2 ATM improves pathological damage of the pancreas and liver in db/db mice

**FIGURE 2:**
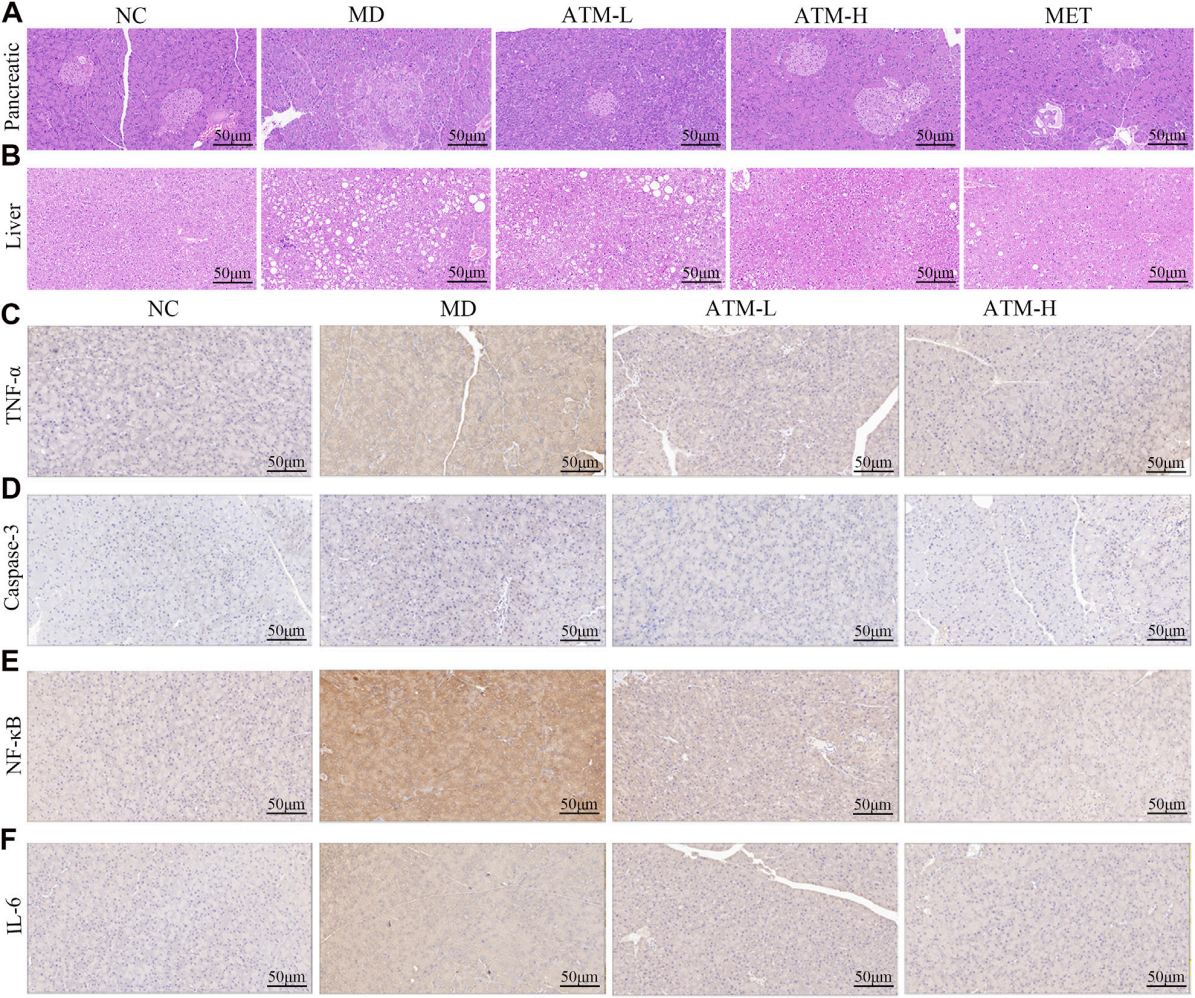
Pancreatic and liver pathology and immunohistochemical images of various inflammatory factors in mice. **(A, B)** Representative H&E staining images of pancreas and liver (200×). **(C–F)** Representative immunohistochemical images of TNF-α, caspase-3, NF-κB, and IL-6 in the pancreas.

Pathological alterations in the pancreas and liver significantly influence the progression of T2DM. The volumetric and structural modifications in the pancreas are associated with insulin secretion and lipid content, whereas the structural and functional variations in the liver are intricately linked to glucose homeostasis and insulin sensitivity. H&E staining of the pancreas showed that pancreatic cell morphology was normal and the islets of the NC group had regular shapes and oval shapes, which were abundantly distributed and evenly arranged (Figure 2A). Nevertheless, the MD group appeared an anomalous pancreas structure, unclear boundary and irregular islet shape, a small number of acinar cells entering the islets, and mild lymphocyte infiltration around the local ducts. Compared to the MD group, the islet morphology of the ATM-L group was partially ameliorated, and the ATM-H group showed arresting improvement in pancreas cytopathy, with clear islet boundary and no obvious inflammatory cell infiltration, which was similar to the islet morphology of NC group. HE staining of the liver showed that a small amount of hydrogenic degeneration and swelling of hepatocytes were observed in the liver tissue of the NC group (Figure 2B). The MD group appeared severely hydropic and ballooning degeneration, prominent presence of fat droplet vacuoles in cytoplasm, and rare lymphocyte infiltration, which were dramatically improved by ATM-H treatment. Moreover, ATM-L treatment markedly decreased the serum ALT level (Figure 1I, *P* < 0.05), indicating that ATM can alleviate liver injury caused by diabetes.

### 3.3 ATM alters inflammatory factors in the serum and tissues of db/db mice

**FIGURE 3:**
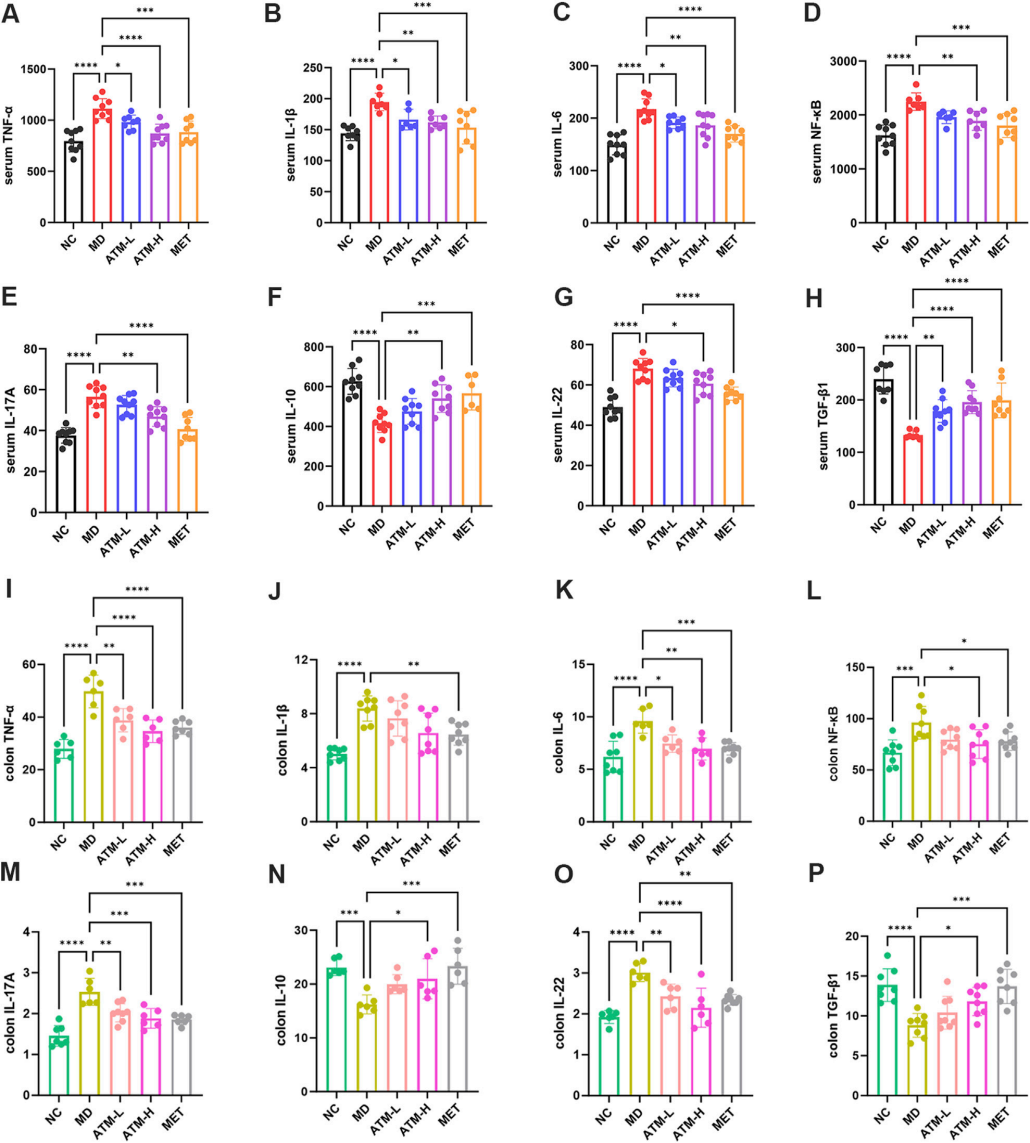
Expressions of inflammatory factors in serum and tissues. **(A–H)** Serum levels of TNF-α, IL-1β, IL-6, NF-κB, IL-17A, IL-10, IL-22 and TGF-*β1* in each group of mice (pg/mL). **(I–P)** Levels of TNF-α, IL-1β, IL-6, NF-κB, IL-17A, IL-10, IL-22 and TGF-*β1* in mice colon tissue of each group (pg/mg protein). *P < 0.05, **P < 0.01, ***P < 0.001, ****P < 0.0001.

A substantial body of evidence indicates that TNF-α, IL-1β, IL-6, NF-κB, IL-17A, IL-10, IL-22, TGF-β1, and LPS are critical inflammatory mediators intricately linked to the inflammatory response. Caspase-3 is the most important terminal cleavage enzyme in apoptotic and modulate the inflammatory response through interactions with inflammatory mediators. The findings of our study indicated that, compared to the NC group, serum levels of TNF-α, IL-1β, IL-6, NF-κB, IL-17A and IL-22 of the MD group were prominently raised, while the expressions of IL-10 and TGF*-β*1 were significantly reduced (Figures 3A–H, *P* < 0.05). Interestingly, ATM-H dramatically recovered the levels of all detected inflammatory factors, and ATM-L induced callbacks of TNF-α, IL-1β, IL-6 and TGF-*β*1 (Figures 3A–H, *P* < 0.05). The colon levels of NF-κB (*P* < 0.001), TNF-α, IL-6, IL-17A, and IL-22 (all *P* < 0.0001) in the MD group were remarkably enhanced compared to the NC group, along with a significant reduction of TGF-*β1* (*P* < 0.0001) and IL-10 (*P* < 0.001) (Figures 3I–P). The ATM-H treatment significantly recovered the levels of TNF-α (*P* < 0.0001), IL-6 (*P* < 0.01), NF-κB (*P* < 0.05), IL-17A (*P* < 0.001), IL-22 (*P* < 0.0001), IL-10 (*P* < 0.05) and TGF-*β1* (*P* < 0.05), ATM-L treatment memorably reduced the levels of IL-6 (*P* < 0.05), TNF-α, IL-17A, and IL-22 (all *P* < 0.01, Figures 3I–P). The LPS level was prominently raised in db/db mice (Figure 1K, *P* < 0.05), which were reduced considerably by ATM treatment at all dosages (Figure 1K, *P* < 0.05). The pancreatic immunohistochemical results indicated that compared with the NC group, the expression levels of TNF-α, Caspase-3, NF-κB and IL-6 in the MD group showed an upward trend (Figures 2C–F). Compared with the MD group, the expression levels of Caspase-3, NF-κB and IL-6 in the ATM-H group showed a downward trend (Figures 2C–F). The above results suggested severe inflammatory storms may occur in diabetic db/db mice, but ATM can alleviate the inflammatory response caused by T2DM.

### 3.4 Network pharmacological analysis predicts the potential targets

**FIGURE 4:**
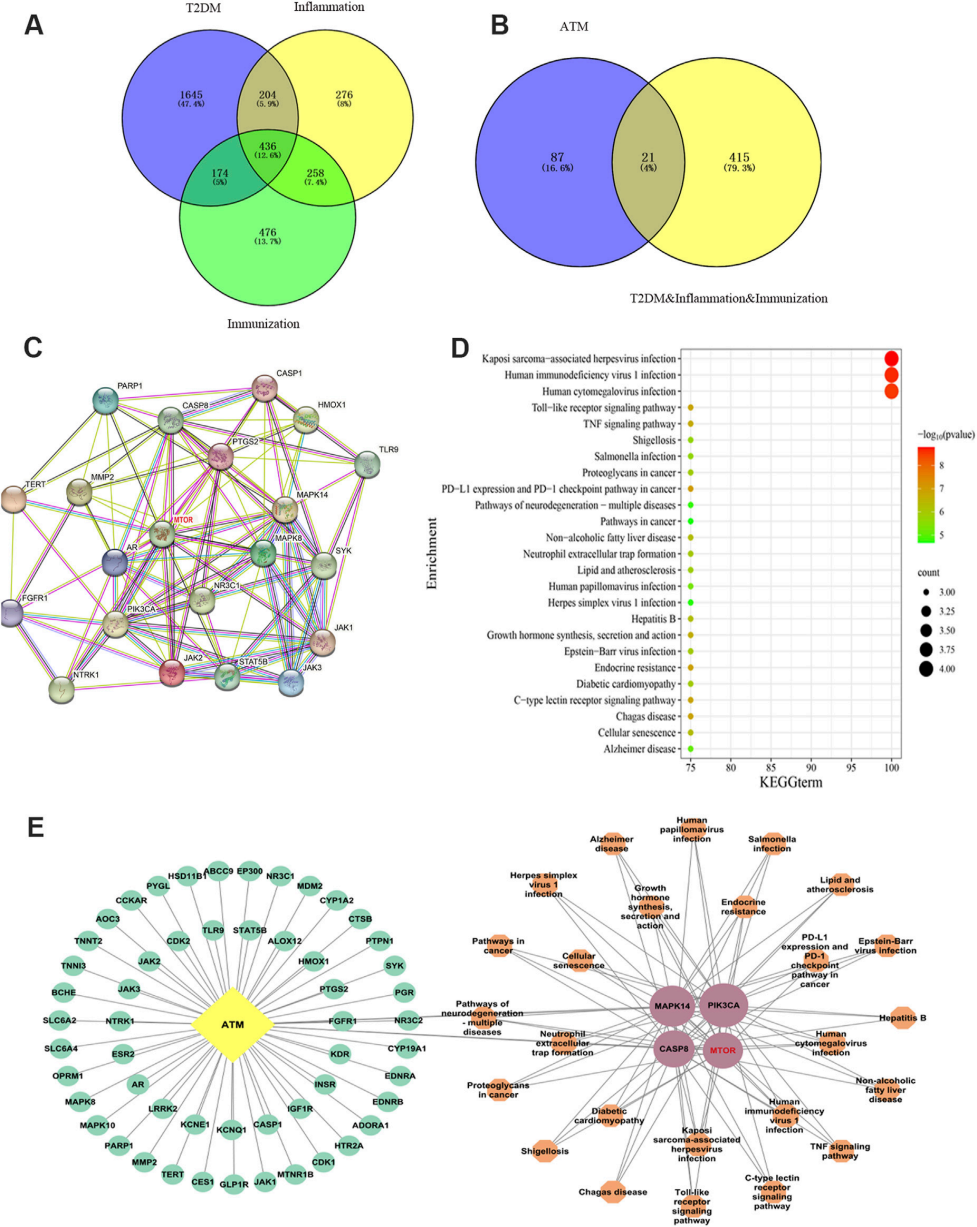
Network pharmacology uncovered the potential mechanism by which ATM may treat T2DM. **(A, B)** Venn diagram screening intersection of ATM, diabetes, inflammation and immune targets. **(C)** PPI network diagram of ATM’s potential targets and core targets in hypoglycemic, anti-inflammatory and immune regulation. **(D)** KEGG enrichment analysis. **(E)** ATM active ingredient-key target-signaling pathway biological network diagram. Yellow represents component ATM, green represents component targets, pink represents key targets, and orange represents key target-related signaling pathways. The larger the shape, the higher the degree value; the smaller the shape, the lower the degree.

Based on TCMSP and databases, 108 targets of ATM were identified, and 436 final targets of T2DM were obtained (Figures 4A, B). After taking the intersection, 21 intersection targets were finally obtained, which may be the potential targets of ATM in hypoglycemic, anti-inflammatory and immune regulation. PPI protein interaction analysis found that the top four core targets and corresponding degree values were mTOR (14), MAPK14 (12), PIK3CA (12) and CASP8 (12), respectively (Figure 4C; Supplementary Figure S1). The mechanism underlying KEGG’s prediction and treatment of T2DM by ATM may be associated with 25 signaling pathways (*P* < 0.01), with the Toll-like receptor signaling pathway and TNF signaling pathway being among the top five representative pathways (Figure 4D). According to KEGG analysis, the ATM active ingredient-key target-signaling pathway network was also constructed (Figure 4E). Altogether, these findings suggested that ATM may reverse glycolipid metabolism back to homeostasis in T2DM through synergistic interaction of all the above targets, especially in mTOR pathways.

### 3.5 ATM improves the disorder of liver metabolites in db/db mice

**FIGURE 5:**
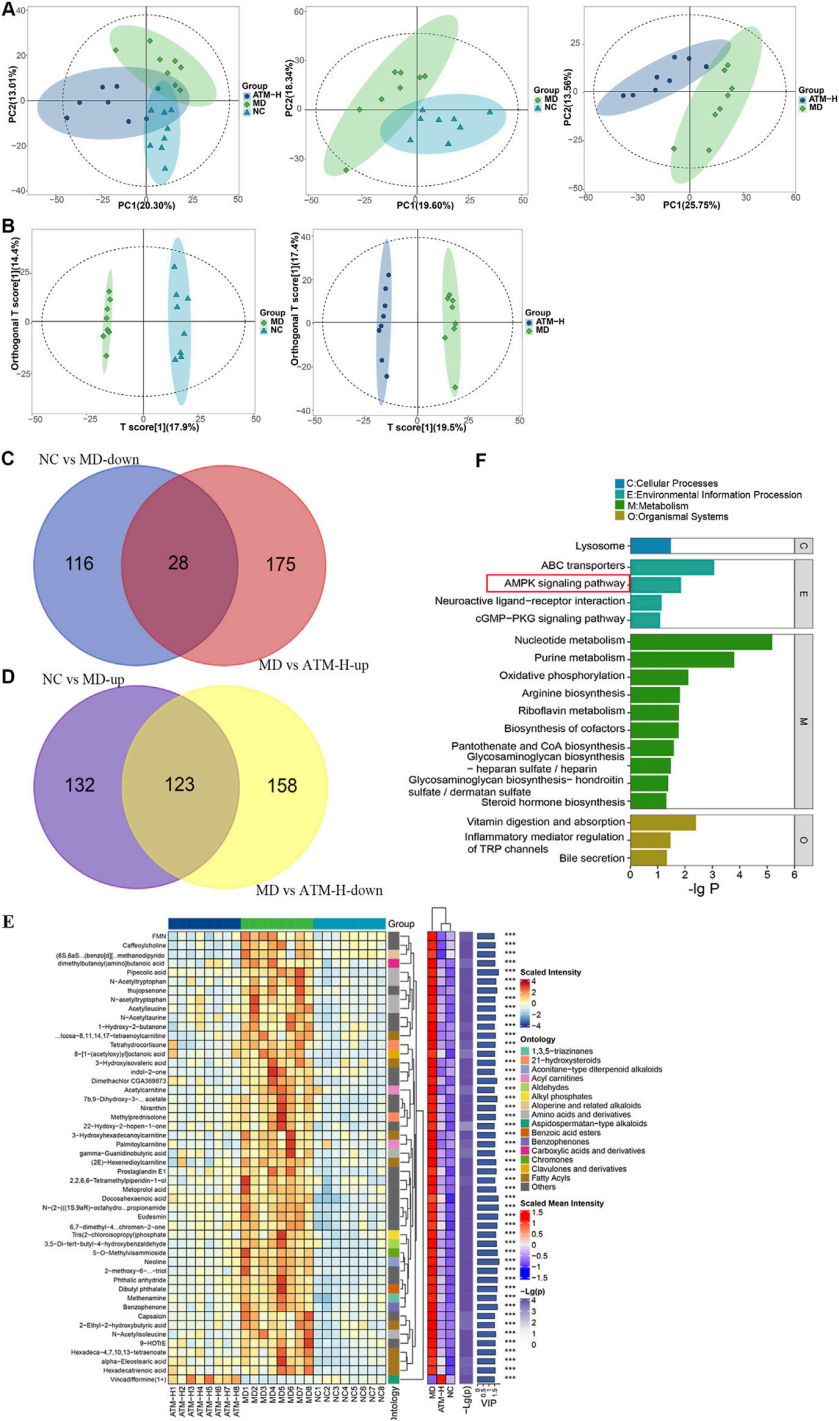
Changes of metabolic level in diabetic mice liver after ATM intervention and analysis of related signaling pathways. PCA **(A)** and OPLS-DA **(B)** scores of NC, MD and ATM-H groups, NC and MD groups, and MD and ATM-H groups, respectively. **(C, D)** Venn diagram screening for up and downregulated metabolites: Compared to the NC group, 28 downregulated metabolites in the MD group were upregulated by ATM **(C)**; Compared with the NC group, 123 upregulated metabolites in the MD group were downregulated by ATM **(D)**. **(E)** Distribution heat map of the differential metabolites. **(F)** KEGG enrichment analysis.

To study the ameliorating effect and potential pathways of ATM on metabolic disorders in T2DM, mice livers of the NC, MD and ATM-H groups were assigned to metabolomics analysis, and the obtained data were evaluated using Principal Component Analysis (PCA) and Orthogonal Partial Least Squares Discriminant Analysis (OPLS-DA) algorithms. PCA is a statistical method for data reduction and variable transformation. It converts multiple correlated variables into a set of linearly uncorrelated principal components through linear transformation. These principal components can retain as much information as possible, and reduce the dimension of the data, which is convenient for analysis and visualization. OPLS-DA is a derivative of PLS-DA and is mainly used in supervised learning scenarios. It combines the methods of orthogonal signal correction and PLS-DA and is able to distinguish between variables that are relevant to the classification information and those that are not. PCA results showed that the NC, MD and ATM-H groups had a high degree of aggregation within each group and could be separated well between the groups (Figure 5A). To identify the vital metabolites leading to this obvious difference, the relevant OPLS-DA analysis was performed to analyze the differential metabolism among the NC, MD and ATM-H groups (Figure 5B). In-depth analysis results presented that the established OPLS-DA models were stable and can be applied for further data analysis (Supplementary Table S1; Supplementary Figure S2) (Liu et al., 2020). A total of 695 different metabolites were screened (*P* < 0.05, VIP ≥ 1). Compared to the NC group, there were 399 different metabolites in the MD group, among which 255 were obviously upregulated and 144 were obviously downregulated. However, compared with the MD group, there were 484 differential metabolites in the ATM-H group, 203 metabolites were remarkably upregulated, and 281 metabolites were dramatically downregulated. Intersection screening of the upregulated and downregulated differential metabolites was performed based on the Draw Venn Diagram platform. 151 Differential metabolites capable of callback after ATM treatment were obtained (Figures 5C, D; Supplementary Table S2, *P* < 0.05). The heat map presented the differential levels of the top 50 differential metabolites among the three groups, which were remarkably increased or decreased in the MD group compared to the NC group, while were reversed to some extent under ATM treatment (Figure 5E, *P* < 0.001). KEGG pathway analysis was performed based on these 151 ATM-capable differential metabolites to explore the underlying mechanisms. A total of 18 significant differential metabolic pathways were identified, among which nucleotide metabolism and purine metabolism were the TOP two pathways related to metabolism. Moreover, the AMPK pathway was the representative pathway related to signal transduction (Figure 5F, *P* < 0.05). The metabolites enriched in nucleotide metabolism and purine metabolism pathways that were reversed significantly by ATM were hypoxanthine, adenosine, inosine, uric acid, UMP, hypoxanthine, adenosine 3′,5′-diphosphate and uridine. The metabolites enriched in the AMPK signaling pathway were NAD and quercetin. These results hinted that ATM could reverse the systemic metabolic disorders induced by glucolipid abnormality in db/db mice through the AMPK pathway.

### 3.6 ATM regulates gut microbiota and enhances SCFAs in db/db mice

**FIGURE 6:**
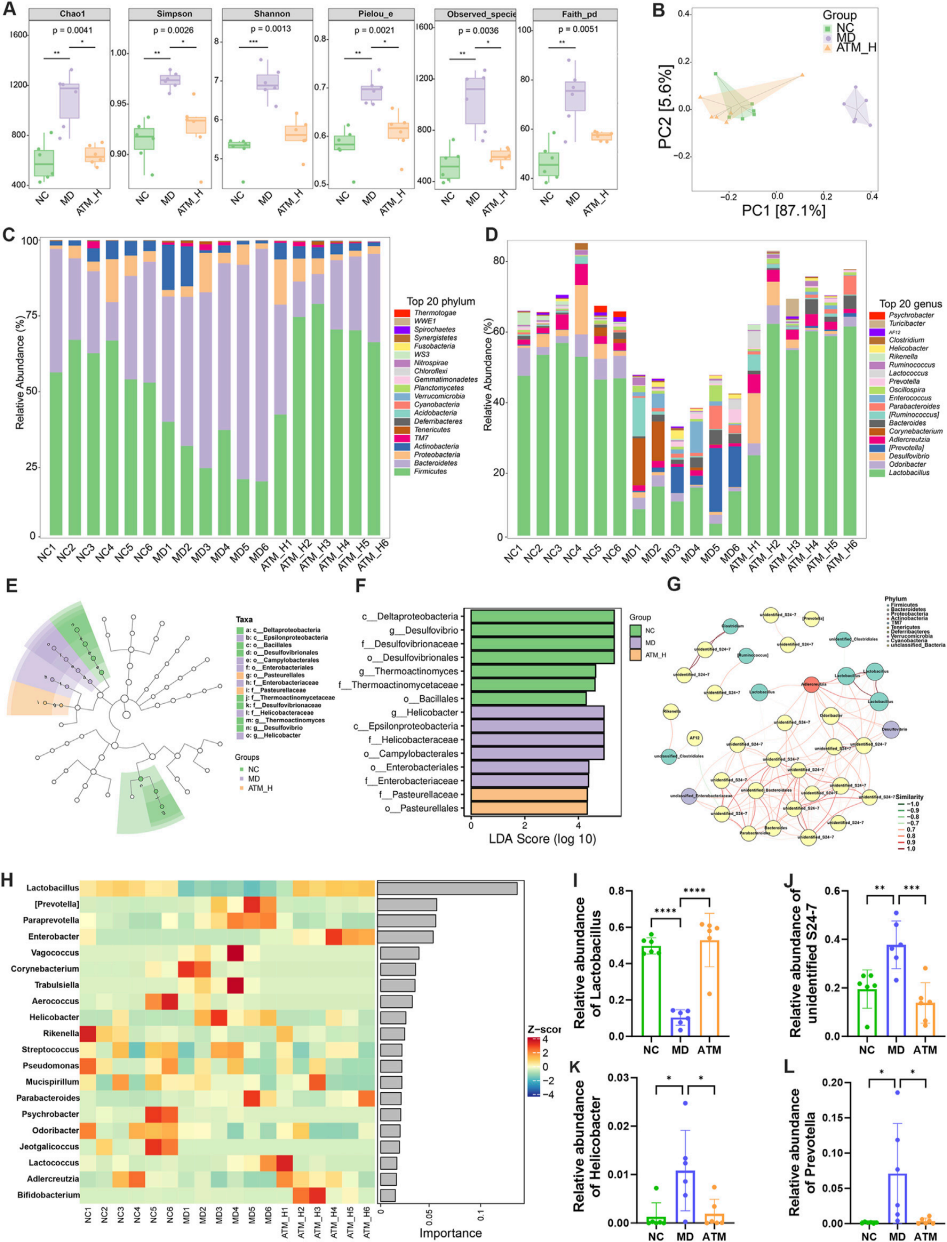
Changes of intestinal flora in mice of NC, MD and ATM-H groups. **(A)** Chao, Shannon, Simpson, Pieiou, Observice-species, Faith index showed α diversity of intestinal flora. **(B)** PCA diagram and Bray Curtis-based PCoA and NMDS diagram. **(C)** The average abundance of the top 20 phylum. **(D)** The relative abundance of the top 20 genera. **(E)** LEfSe analysis of the evolutionary branching diagram. **(F)** LDA effect size shows the most obvious differential microbiota among the three groups. **(G)** Network plot of the top 50 dominant species for average abundance annotated at the phylum level. **(H)** Bacterial heat maps of the top 20 importance of Random Forests at the genus level. **(I–L)** Relative abundance of *Prevotella, Lactobacillus, unidentified S24-7 and Helicobacter*.

To clarify the influence of ATM on the gut microbiome, the composition of that was analyzed in db/db colon feces mice by 16S rRNA technology. The microbial diversity of the MD group was remarkably higher compared to the NC group, however, the microbial diversity of the MD group could be reversed by ATM-H treatment (Figure 6A, *P* < 0.05). The composition of gut microbiota was also obviously different among the NC, MD, and ATM-H groups (Figure 6B). *Firmicutes* was the dominant phylum in the NC group with *Bacteroidetes* in the MD group, while ATM-H treatment re-constructed the gut microbiota composition by enhancing the abundance of *Firmicutes* and reducing the abundance of *Bacteroidetes* (Figure 6C). At the genus level, the *Lactobacillus* abundance was observably increased and *Prevotella* significantly decreased after ATM treatment (Figure 6D). Lefse analysis was used to screen out 15 different microorganisms based on *P* < 0.05 and LDA > 4 criteria, among which 7 were enriched in NC group, six in MD group, and two in the ATM-H group (Figures 6E, F). *Desulfovibrio* was the predominant representative microbe in the NC group, while *Helicobacter* was the top representative microbe in the MD group. The subnetwork map of the dominant species showed that the dominant bacteria with the most nodes were *unidentified S24-7* and *Lactobacillus* (Figure 6G). According to Random Forests analysis, the top two important microorganisms in the genus level were *Lactobacillus* and *Prevotella* (Figure 6H). Finally, the *Lactobacillus*, *unidentified S24-7*, *Helicobacter*, and *Prevotella* microbes (the relative abundances shown in Figures 6I–L) were chosen for subsequent co-occurrence analysis.

**FIGURE 7:**
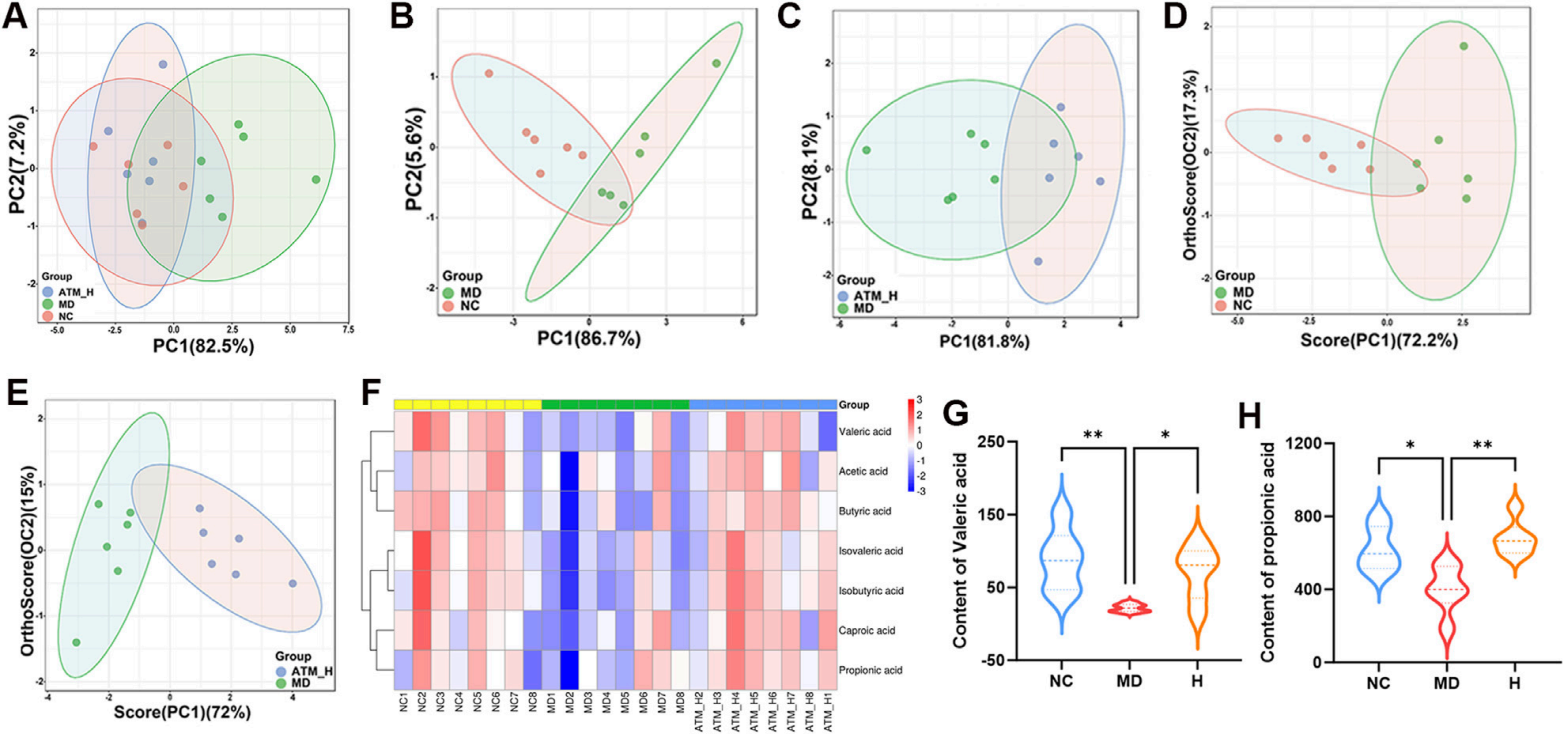
Changes of SCFAs levels of mice in each group. **(A–C)** PCA score plots between the MD, NC and ATM-H groups, the NC and MD groups, the MD and ATM-H groups **(D, E)** OPLS-DA score plots between NC and MD groups, MD and ATM-H groups. **(F)** Heat map of SCFAs. **(G)** The level of propionic acid. **(H)** The level of valerate acid. *P < 0.05, **P < 0.01.

The colon feces of mice were selected for targeted analysis of gut SCFAs. According to PCA analysis, gut SCFAs varied among three groups (Figures 7A–C). In OPLS-DA analysis, gut SCFAs of healthy mice were significantly separated from db/db mice, which were obviously separated from mice of the ATM-H group, indicating significant changes in gut metabolites of db/db mice compared with NC and ATM-H mice (Figures 7D, E). The levels of all SCFAs in each group are shown in Figure 7F, indicating that SCFAs metabolism is disrupted in diabetic mice, a certain degree of regression can be demonstrated after intervention with ATM. In addition, SCFAs with *P* < 0.05 and VIP ≥ 1 were selected for further analysis, and two differential metabolites were screened out, propionic acid and valeric acid. The levels of propionic acid and valeric acid in the MD group were significantly lower than those in the NC group, and the levels of propionic acid (*P* < 0.01) and valeric acid (*P* < 0.05) in the ATM-H group were significantly higher than those in the MD group (Figures 7G, H). These results indicated that ATM-rebalanced systemic glucolipid metabolic disorders depended on regulating gut microbial dysbiosis and SCFAs, especially propionic and valeric acids.

### 3.7 ATM affects the expression of the vital proteins in the AMPK/mTOR pathway

The AMPK/mTOR pathway was identified as the main signaling pathway by metabolomics and network pharmacology. The related targets screened were verified to confirm whether ATM acted on this pathway. WB method was used to detect the levels of pathway-associated proteins p-AMPK, p-mTOR, p-P38, p-P65, p-IKB*α*, p-IRS1 and TLR4 in the mice liver and colon. The proteins ZO-1, occludin, and claudin-1 are crucial components of the intestinal tight junction complex, playing a pivotal role in maintaining the integrity of the intestinal barrier and regulating its permeability. These proteins effectively seal intercellular spaces by forming tight junction complexes, thereby preventing the leakage of harmful substances while selectively allowing the passage of nutrients and waste products. Consequently, the protein expression levels of ZO-1, occludin and claudin-1 in the mouse colon were also investigated.

**FIGURE 8:**
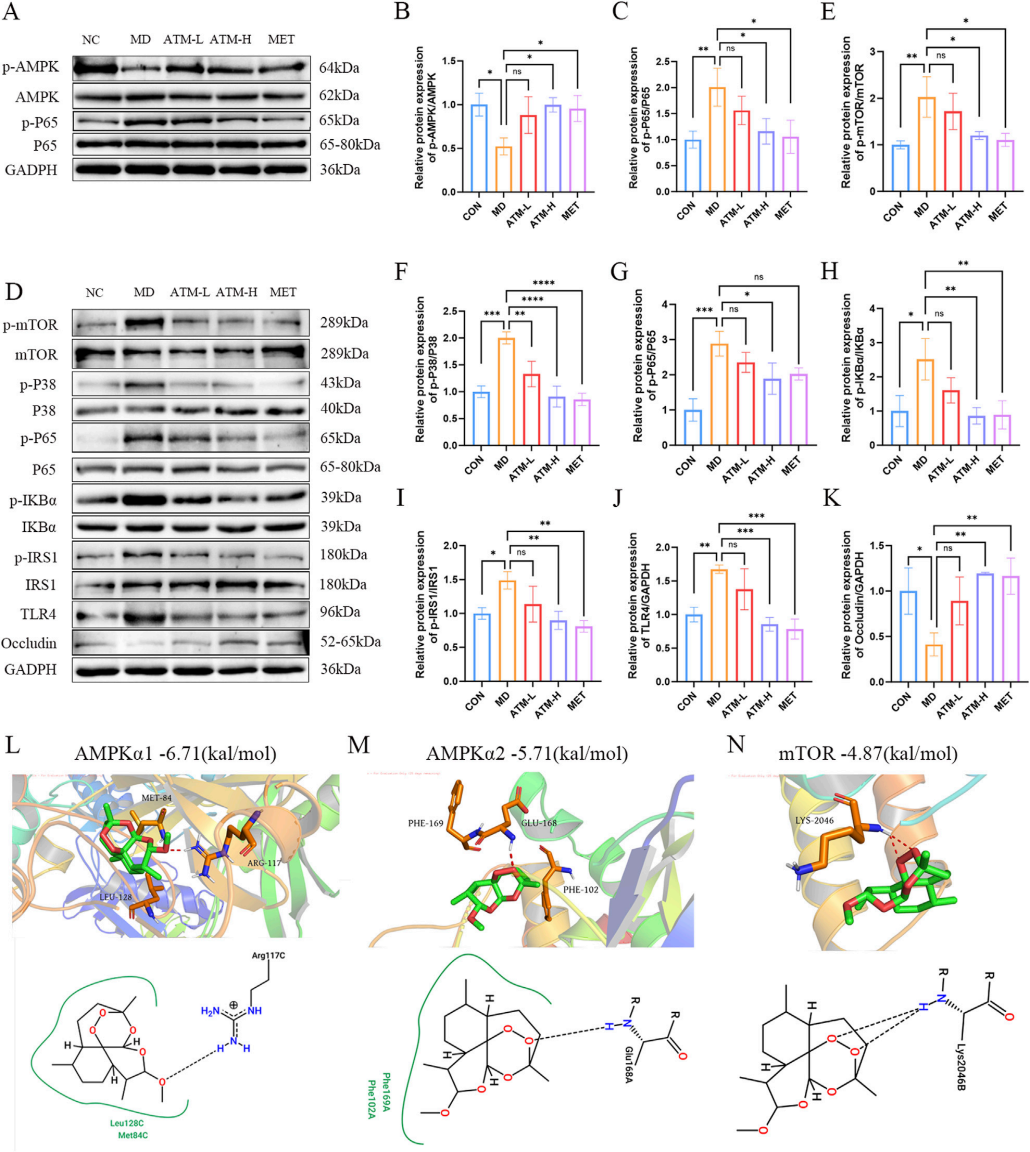
Relative expression of AMPK/mTOR pathway-related proteins and molecular docking. **(A–C)** The levels of p-AMPK and p-P65 proteins in liver tissue. **(D–K)** The relative expressions of p-mTOR, p-P38, p-P65, p-IKB*α*, p-IRS1, TLR4 and occludin proteins in colon tissues. **(L–N)** The molecular docking results of ATM with AMPK*α*1 (PDB ID: 6C9H), AMPK*α*2 (PDB ID: 3AQV), and mTOR (PDB ID: 1AUE) are presented below. *P < 0.05, **P < 0.01, ***P < 0.001, ****P < 0.0001.

For the liver tissues, compared with NC group, the protein levels of p-P65 (*P* < 0.01) of MD group were dramatically enhanced, and p-AMPK expression were impaired (Figures 8A–C). Moreover, the expression of p-mTOR, p-P38, p-IKBα, p-IRS1, TLR4 showed an increasing trend without no significant difference among three groups (Supplementary Figures S3A–E). ATM-H treatment obviously reduced the protein expression of p-P65 (Figure 8C, *P* < 0.05). In addition, ATM-H treatment also increased the level of p-AMPK (Figure 8B, *P* < 0.05).

In the colon tissue, compared with NC, the protein levels of p-mTOR, p-p38, p-IKB*α*, TLR4, p-p65 and p-IRS1 of the MD group were obviously increased, which were reduced considerably after ATM-H treatment (Figures 8D–J, *P* < 0.05). Moreover, the intestinal permeability protein levels of ZO-1, occludin and claudin-1 in the MD group was obviously reduced (Figure 8K; Supplementary Figures S3G, H, all *P* < 0.05). Compared with MD group, the levels of occludin in the ATM-H group was obviously increased (Figure 8K, *P* < 0.01), and the levels of ZO-1 and claudin-1 protein showed an upward trend in all ATM groups (Supplementary Figures S3G, H). The above results suggested that the target proteins of the AMPK/mTOR pathway were inhibited to a certain extent after ATM treatment, and the intestinal permeability-related proteins occludin were significantly increase after ATM treatment.

### 3.8 Molecular docking results

To investigate the regulatory impact of ATM on key targets within the AMPK/mTOR pathway, we performed molecular docking simulations between ATM and AMPK (*α*1 and *α*2 isoforms) as well as mTOR (Figures 8L–N). Our results indicate that the binding energies of ATM with AMPK*α*1, AMPK*α*2, and mTOR are −6.71 kcal/mol, −5.71 kcal/mol, and −4.87 kcal/mol, respectively. Notably, the interaction between ATM and AMPK*α*1 exhibits greater stability and stronger affinity compared to the other targets.

### 3.9 Co-occurrence analysis of gut bacteria, metabolites and proinflammatory cytokines

**FIGURE 9:**
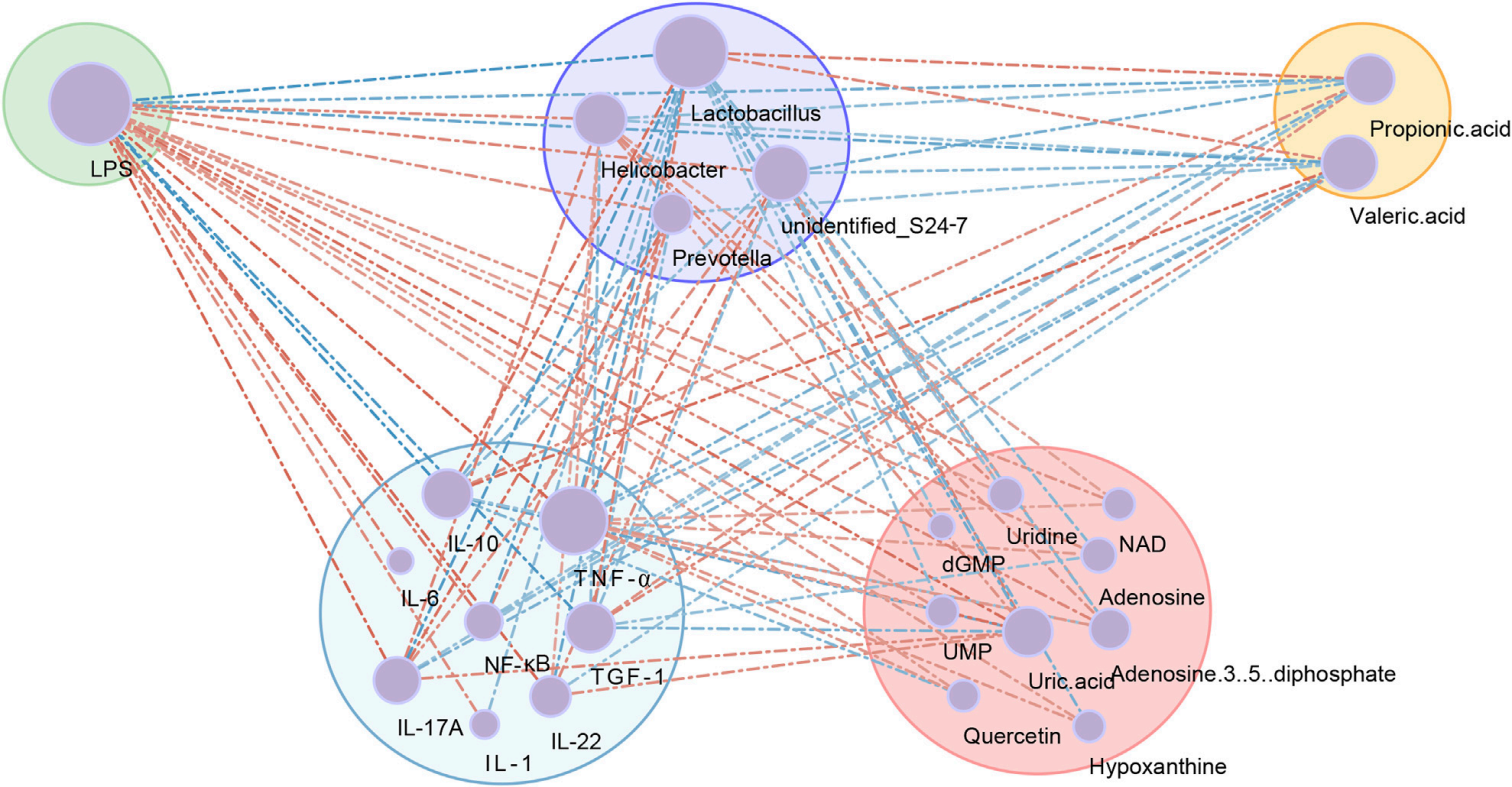
Co-occurrence network showing the correlation values among differential gut microbial genera, proinflammatory cytokines and metabolites. The node size indicated the degree of the above variables. Lines indicated Spearman’s positive (red) or negative (blue) correlations.

**FIGURE 10:**
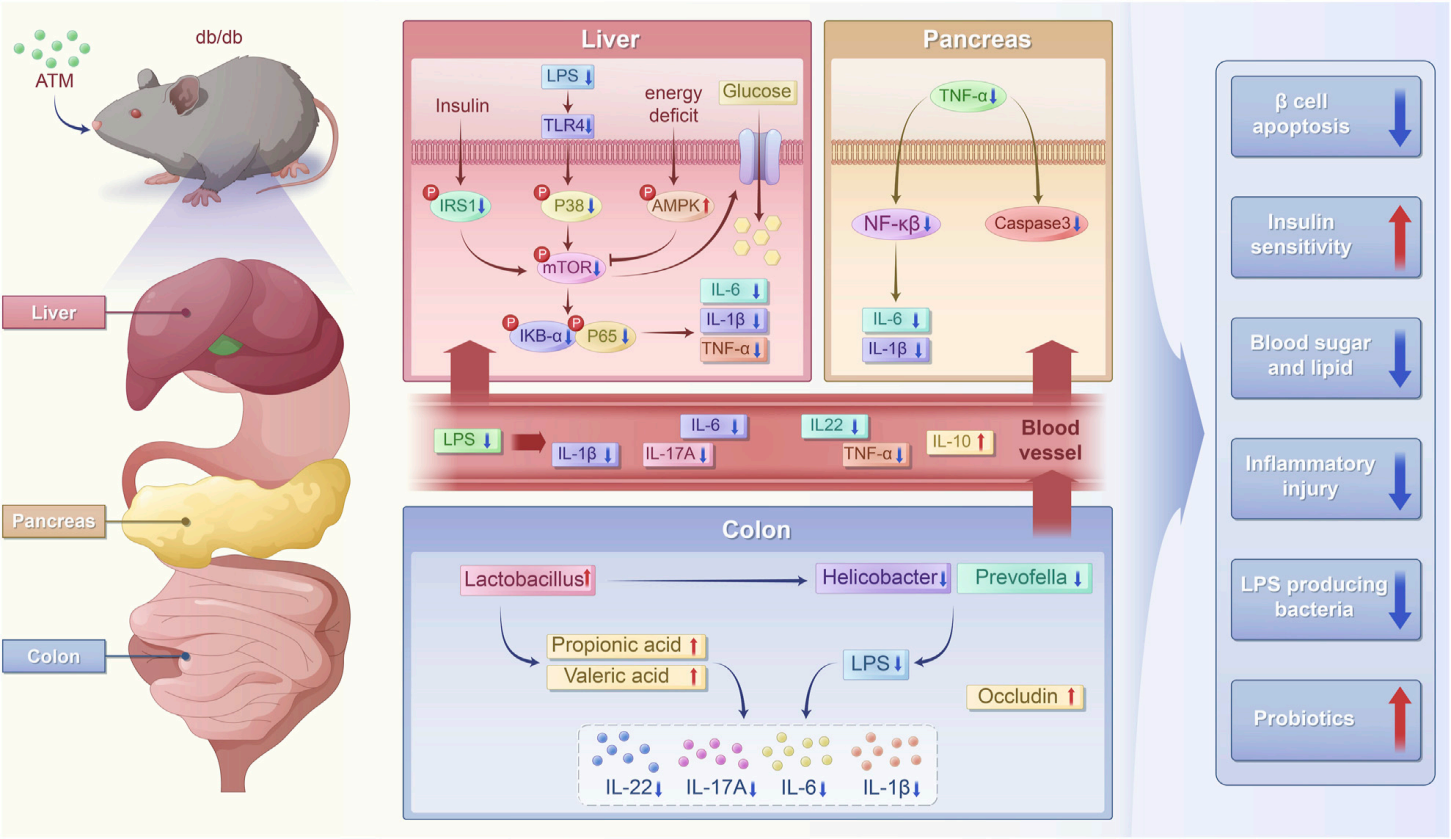
Schematic chart of the potential mechanism of ATM ameliorating glucose and lipid metabolic disorders in T2DM.

To further understand the mechanism of ATM in T2DM, spearman correlation analysis of gut bacteria, proinflammatory cytokines and metabolites was performed. Four differential microbiota, nine differential nucleotide and purine pathway metabolites, eight differential serum proinflammatory cytokines, two differential SCFAs and the LPS were included. A network diagram was constructed (Figure 9). There exist eight major interconnected clusters, including LPS, *Lactobacillus*, propionic acid, TNF-α, IL-10, IL-17A, NF-κB and uric acid. Few key elements emerged. For instance, LPS was positively correlated with TNF-α, IL-17A, NF-κB, *Helicobacter*, *Prevotella*, uric acid, and inosine, and negatively related to IL-10, propionic acid, and valeric acid. *Lactobacillus* was negatively correlated with LPS, *Prevotella*, *Helicobacter*, adenosine, hypoxanthine and inosine, and positively correlated with propionic acid and valeric acid. TNF-α was positively correlated with IL-1β, IL-6, NF-κB, IL-17A, *Helicobacter*, *Prevotella*, adenosine, and hypoxanthine, and negatively correlated with IL-10, propionic acid and valeric acid. According to the interconnected network results, these factors with complex associations may contribute to the mechanism of ATM’s action in T2DM (Figure 10).

## 4 Discussion

T2DM is a common metabolic disease in the world and has become a global health problem due to rising morbidity and mortality (Zhou et al., 2022). Artemether has previously exhibited the beneficial influence of glucose and lipid metabolism in T2DM mice, like ameliorating glucose homeostasis and IR, preventing fatty liver and obesity, and protecting pancreatic *β*-cells (Guo et al., 2018; Bai et al., 2020). Nevertheless, the molecular mechanism by which ATM exerts these functions and what kind of role the gut flora plays remain unclear. This study aims to explore how ATM alleviates glycolipid metabolism and inflammatory stress in db/db mice and investigate its diabetes-related mechanisms in the pancreas, liver and intestine of mice by co-integrated analysis of metabolomics, microbiome and network pharmacology to provide evidence for its rational application in T2DM.

### 4.1 Pharmacodynamic evaluation of ATM for the improvement of metabolic disorders in T2DM

In this study, db/db mice exhibited severe glucose metabolism disorders, including increased water intake, diet intake, body weight, random blood glucose, FBG, and FINS, which were significantly reversed by ATM treatment. IPGTT and IPITT results revealed that ATM effectively decreased blood glucose and enhanced insulin sensitivity in DM mice. DM cases often exhibit disorders of lipid metabolism, which are seen as dyslipidemia. Patients with diabetes usually have accelerated lipolysis, resulting in elevated serum TC and TG levels (Zhou Z. W. et al., 2023). In this study, db/db mice displayed severe lipid metabolism dysfunction with arresting increased TG level, which was significantly reduced by ATM treatment. These results indicated that ATM could adjust the lipid metabolism in db/db mice, thereby partially alleviating the progression of diabetes. Furthermore, ATM treatment reduced serum ALT levels and alleviated hepatocyte damage in diabetic mice. Further studies showed that db/db mice had some pathological damage to the pancreas and liver with pancreatic tissue changes, irregular islet shape, unclear boundary, a small number of acinar cells into the islet, a small amount of lymphocyte infiltration, extensive liver cell hydropathy and ballooning degeneration, cytoplasmic vacuolization, and a large number of hepatocyte steatosis. After 160 mg/kg of ATM treatment, the pancreas and liver injury were improved. Besides, the 160 mg/kg ATM was better than the 80 mg/kg ATM. Recent studies have demonstrated that ATM regulates liver glycogen and lipid utilization through mitochondrial pyruvate oxidation in db/db mice (Weng et al., 2024). In addition, Bai et al. discovered that ATM modulates the expression of enzymes associated with glucose and lipid metabolism in the livers of db/db mice (Bai et al., 2020). In our previous study, intervention with ATM (200 mg/kg) for 2 weeks significantly improved blood glucose homeostasis and insulin resistance in db/db mice, and had the effect of preventing obesity and reducing fatty liver (Guo et al., 2018). The above findings align with the outcomes of our present indicating that ATM has the potential to improve glucolipid metabolism, alleviate IR, and lower blood glucose levels in T2DM.

### 4.2 Effect of ATM on the inflammatory state of T2DM

Recently, it has become clear that T2DM exhibits the feature of chronic metabolic inflammation (Scheithauer et al., 2020), which has been identified to be highly related to the pathogenesis of diabetes (Saad et al., 2016). To further investigate the relationship between ATM and inflammation, the levels of inflammatory factors in both the serum and tissues of mice were assessed. As known, LPS disrupts glucose tolerance by inducing IR and hindering insulin expression (Cani et al., 2007). LPS can bind to TLR4, induce mTOR phosphorylation, and activate the classical NF-κB pathway (Zhou et al., 2018), thus inducing systemic inflammation and oxidative stress (Ghoshal et al., 2009). In this study, LPS levels were significantly increased in db/db mice, but reduced by ATM at all doses. Moreover, ATM dramatically reduced the expressions of TNF-α, IL-1β, IL-6, NF-κB and IL-17A, and significantly enhanced the level of IL-10 in diabetic mice. TNF-α, IL-1β, IL-6, NF-κB and IL-17A are multifunctional cytokines, that are mainly related to immune regulation, and play a crucial role in the occurrence and progression of diabetes (Dror et al., 2017; Sigurdardottir et al., 2019). Some studies have reported that T2DM patients have lower IL-10 expression and higher proinflammatory signals (van Exel et al., 2002; Lumeng et al., 2007). Overexpression of IL-10 in mice muscle tissue can improve insulin sensitivity (Hong et al., 2009). The immunohistochemistry results showed that the pancreatic levels of TNF-α, IL-6, NF-κB and caspase-3 were significantly decreased after ATM intervention. The activation of caspase-3 may induce islet *β* cell apoptosis in T2DM patients, which may be an important pathophysiological mechanism of T2DM (Sun et al., 2021). These findings suggested that ATM may treat T2DM by alleviating the inflammatory state.

### 4.3 Effects of ATM on gut microbiota and SCFAs in T2DM

The gut microbiota plays a crucial role in the pathogenesis and progression of T2DM, and alterations in its community composition and metabolites are intricately linked to the pathophysiological processes of T2DM. Gut microbes regulate inflammation and interact with dietary components to affect intestinal permeability, glucolipid metabolism, insulin sensitivity, as well as overall energy homeostasis in mammalian hosts (Aw and Fukuda, 2018). Therefore, this study aims to explore the potential mechanism of gut microbiota in ATM treatment of T2DM. In this research, 16S rRNA was applied to explore the ATM’s influence on the composition of the gut microbiota of all groups. *Firmicutes* were the key phylum in the NC group, and *Bacteroidetes* were the dominant phylum in the model group. ATM rebalanced gut microbial dysbiosis by decreasing the abundance of *Bacteroidetes* and increasing the abundance of *Firmicutes*. In accordance with our results, Larsen et al. found an obvious reduction in the proportion of *Firmicutes* and a slight increase in the proportion of *Bacteroidetes* in the intestine of diabetic patients, and the ratio of *Bacteroidetes* to *Firmicutes* was positively associated with impaired glucose tolerance (Larsen et al., 2010). Some gut microbial products, especially LPS, accelerate metabolic endotoxemia and inflammation by stimulating inflammatory cytokines (Gurung et al., 2020). LPS was significantly increased in db/db mice in this study, which may be related to the increase of *Helicobacter* and *Prevotella* in the gut microbiome. After ATM intervention, the abundance of *Helicobacter* and *Prevotella* significantly decreased, and the LPS level also observably decreased. *Helicobacter pylori* resides in the gastric antrum and secretes cytotoxins thus causing inflammation and immune response. Multiple studies have found that *H. pylori* infection is related to various extra gastric diseases, like cardiovascular disease and dementia (Lai et al., 2015), especially IR and diabetes (Jeon et al., 2012; Gravina et al., 2018; Jeon et al., 2012). It is believed that *H. pylori* infection causes higher incidence of diabetes, and antibiotics and gastrointestinal treatment can prevent the development of diabetes (Jeon et al., 2012). *Prevotella* genus is an anaerobic bacterium of *Bacteroidetes*, which can induce epithelial cells to release IL-8 and IL-6, and facilitate mucosal immune responses and neutrophil recruitment. Studies have found that an increased abundance of *Prevotella* correlates highly with metabolic disorders and a systemic low-inflammatory state (Larsen, 2017). Therefore, ATM may regulate the inflammatory state in diabetic mice by reducing the abundance of *Helicobacter* and *Prevotella*. In addition, the abundance of *Lactobacillus* and *unidentified S24-7* was obviously decreased in db/db mice and could be enhanced by ATM treatment. *Lactobacillus* is one of the most studied probiotics inhabiting the human body and plays a beneficial role by inhibiting the growth of pathogenic microorganisms, keeping the local microenvironment balance (Kim and Woo, 2014). *Lactobacillus* plays an anti-inflammatory role in macrophages through MAPK and NF-κB signal transduction through ketone bodies produced by the polyunsaturated fatty acid pathway (Sulijaya et al., 2020). In addition, *ketoacidophilus* produced by *Lactobacillus* inhibits the production of IL-6, IL-1β and TNF-α (Sulijaya et al., 2020). Thus, it can reduce the inflammatory state, reduce blood glucose, and improve IR in diabetic patients (Luoto et al., 2010; Moroti et al., 2012). It was found that barley intake could have beneficial effects on db/db mice by increasing the relative abundance of S24-7 (Garcia-Mazcorro et al., 2018).

SCFAs are generated by the intestinal microbiota via the anaerobic fermentation of dietary fiber and resistant starch. SCFAs play a crucial role in maintaining host health by modulating intestinal barrier function, microbial activity, and mechanisms associated with glucose homeostasis (Chambers et al., 2018). To deeply understand the effects of ATM on gut microbial metabolites, targeted metabolomics analysis of SCFAs was performed on the colon feces of db/db mice. Compared to the MD group, ATM could significantly increase the content of SCFAs, particularly propionic acid and valeric acid. The increased SCFA levels in the ATM-H group may associated with the increased abundance of *Lactobacillus* and other bacterial groups producing SCFA in large amounts (Bishehsari et al., 2018; Chen and Zhang, 2023). Propionate can suppress intracellular lipolysis, improve the lipid buffering capacity of adipose tissue, reduce lipid deposition, and enhance insulin sensitivity (Zhang Z. et al., 2019). Additionally, Yibing Zhou et al. (Zhou Y. et al., 2023) reported that valerate can increase the concentration of GPR43 in the colon, which in turn reduces the expression of the NLRP3 inflammasome, TNF-α, and IL-6, thereby decreasing lipid deposition. In summary, ATM may improve glucolipid metabolism and mitigate inflammation through the regulation of intestinal microbial composition, reduction of LPS production, elevation of SCFAs, particularly propionate and valerate, in db/db mice.

### 4.4 Effects of ATM on hepatic metabolic in T2DM

To investigate the mechanisms by which ATM improves glucolipid metabolism in T2DM, we conducted untargeted metabolomics analysis of mice liver tissue. The results revealed that db/db mice had a significant metabolic disorder. KEGG enrichment analysis displayed that nucleotide metabolism and purine metabolism are the two most significantly different metabolic pathways. Metabolites that participate in the regulation of these two pathways and exhibit significant differences include hypoxanthine, adenosine, inosine, uric acid, NAD, dGMP, UMP, adenosine 3′,5′-diphosphate, uridine, and quercetin. Nucleotides are the building blocks of DNA and RNA, which have a variety of intracellular and extracellular signaling functions and exert important functions in the pathophysiology of T2DM (Jorquera et al., 2021; Liu et al., 2022). As an important intermediate metabolite and purine molecule, adenosine is the cornerstone of adenine biosynthesis or ATP formation and has an important relationship with glucose homeostasis and IR (Chen et al., 2013). Extracellular adenosine can induce IR and hyperglycemia and inhibit lipolysis of adipocytes (Liu et al., 2022). Metabolomics data in this research indicated that adenosine levels were obviously increased in db/db mice, but reversed after ATM treatment. Uridine is a simple metabolite involved in nucleic acid synthesis, oxidative stress and inflammatory response. It is essential for glycogen synthesis by forming uridine diphosphate glucose (Roach et al., 2012). In addition, it can be seen that the level of uridine was obviously increased in db/db mice, and the increase of uridine was closely related to glucose homeostasis (Nogal et al., 2023). Uridine supplementation enhances the O-GlcNAc glycosylation levels of insulin receptors, Akt and mTOR, which are the crucial elements of insulin signaling propagation and modification in the liver (Le et al., 2014). O-GlcNAc glycosylation of these components decreases the cellular insulin response, leading to IR (Issad et al., 2010). The intervention of ATM significantly reduced the level of uridine suggesting that ATM can regulate the levels of adenosine, uridine and other nucleotides, thereby reducing hyperglycemia and improving IR. Purines are the basic building blocks of nucleic acids and play many key roles in physiology, affecting tissue function (Varadaiah et al., 2022). Serum uric acid may cause a high risk of peripheral arterial disease, IR, as well as metabolic syndrome (Copur et al., 2022). Elevated purine metabolite levels in diabetic patients are closely related to increased hepatic glucose production and decreased pancreatic insulin secretion (Varadaiah et al., 2022). A clinical study found that diabetic patients had increased dephosphorylation of adenine and guanine, and higher concentrations of adenosine, inosine, and hypoxanthine, which was characterized by severe purine metabolism disorders (Dudzinska, 2014). These results are in line with our findings that db/db mice showed significant changes in purine metabolism in the liver, with obvious increases in the levels of hypoxanthine, adenosine, inosine, uric acid and dGMP, and all recovered by ATM treatment.

Interestingly, among the signal transduction pathways, we found that the AMPK pathway may be a key signaling pathway in ATM’s treatment of T2DM. AMPK is a serine/threonine kinase adjusting body weight and glycolipid metabolism and plays a key role in the progression of diabetes and glycolipid homeostasis (Zhang et al., 2020). Recent studies have shown that *Ficus carica* leaves may reduce apoptosis of *β*-cells by suppressing AMPK activity (Zhang et al., 2020). AMPK also increases the mRNA levels of genes encoding hexokinase two and GLUT4 to promote glucose uptake (Stoppani et al., 2002). Our previous research demonstrated that ATM potentially upregulates GLUT-4 and IR*β* protein expression via the AMPK pathway, consequently ameliorating hyperlipidemia and insulin resistance in diabetic mice (Fu et al., 2020). These are all consistent with our results indicating that AMPK could serve as a pivotal target for ATM in the regulation of metabolic disorders and the mitigation of inflammatory responses in T2DM. In conclusion, ATM may regulates hepatic metabolites through AMPK pathway by improving imbalances of nucleotide and purine metabolism, thereby ameliorating metabolism disorders.

### 4.5 Network pharmacological analysis

To further understand the mechanism of ATM in glucose and lipid reduction, network pharmacology was applied to explore the latent targets of ATM in diabetes. A total of 109 targets corresponding to ATM were obtained through database retrieval. Up to 436 disease targets were identified from the disease database, and 21 intersection targets were obtained after the intersection. Then the 21 intersection targets were applied to establish the PPI network. Notably, mTOR ranked first according to the degree value, which may become the key target of ATM in hypoglycemic, anti-inflammatory and immune regulation. After the onset of diabetes, the levels of human autoinflammatory factors and reactive oxygen species increase, which can activate RTKs and GPCRs, leading to the phosphorylation of mTOR to activate mTOR (Dong et al., 2023). The activation of the mTOR pathway can trigger a series of signaling cascades such as the release of inflammatory cytokines, oxidative stress, apoptosis and autophagy, which will further aggravate the progression of diabetes (Asahara et al., 2022).

The integrated analysis of network pharmacology and metabolomics revealed that the AMPK/mTOR pathway is likely a pivotal mechanism through which ATM exerts its therapeutic effects on T2DM. Specifically, AMPK acts as an upstream regulator of mTOR, directly phosphorylating the raptor protein within the mTOR complex, thereby inhibiting mTORC1 activity. Notably, several studies have demonstrated that SCFAs can activate the AMPK/mTOR signaling pathway via multiple mechanisms, leading to diverse physiological effects. For instance, Tang et al. reported that propionate-induced autophagy is associated with reduced mTOR activity and increased AMP kinase activity (Tang et al., 2011). Propionic acid-induced autophagy stems from mitochondrial dysfunction characterized by cellular ATP depletion and ROS production, both of which promote AMPK activation and subsequent mTOR inhibition. Collectively, these findings suggest that ATM may alleviate inflammation and metabolic disorders in T2DM by modulating gut microbial composition, elevating SCFA levels, and activating the AMPK/mTOR pathway.

### 4.6 Validation of the mechanism of action of ATM in improving T2DM

Relevant targets in the AMPK/mTOR pathway were validated based on the targets and pathways enriched via network pharmacology and metabolomics. mTOR can regulate cell growth, proliferation and apoptosis, and determine cell fate, and the AMPK pathway is a key factor in mTOR signaling through the inhibition of mTORC1 activity by adjusting the activity of the TSC1/TSC2 complex (Maiese, 2016). In this study, AMPK phosphorylation was significantly activated after ATM intervention, while mTOR phosphorylation was inhibited. Moreover, the phosphorylation of IRS1 and P38, the upstream proteins of the mTOR pathway, was significantly inhibited. Molecular docking simulations for AMPK (*α*1 and *α*2 isoforms) and mTOR were performed to predict the binding potential of ATM with the aforementioned target proteins. Encouragingly, ATM exhibited strong binding affinity with both AMPK and mTOR, with AMPK*α*1 demonstrating the highest binding affinity at −6.71 kcal/mol.

Some studies have found that activation of mTOR may regulate intestinal inflammation through the upstream TLR4 and P38 and downstream NF-κB pathways, and inhibition of mTOR expression can significantly inhibit inflammatory response (Zhou et al., 2018). As a receptor for LPS, TLR is highly expressed in the membrane of various epithelial cells. The inhibition of TLR4 and mTOR signaling pathways by L-arginine may attenuate intestinal mucosal destruction (Tan et al., 2014). Activation of the mTOR signaling pathway can upregulate the TLR4 expression, thereby triggering macrophage activation and causing an inflammatory response (Yu et al., 2011). Consistently, TLR4, P38 and IRS1 upstream proteins of mTOR were significantly downregulated, and the critical proteins in the NF-κB pathway downstream of mTOR were significantly inhibited after ATM treatment. The intact intestinal barrier is a crucial defense to stop harmful substances from entering the organisms and ensure the stability of the internal environment (Fan et al., 2021). Occludin, ZO-1, and claudin-1 are typical intercellular tight junction proteins related to intestinal integrity. It is well-documented that elevated intestinal permeability is an important driver of chronic inflammatory responses in diabetic patients (Mishra et al., 2023). In this study, the expressions of occludin, ZO-1 and claudin-1 proteins in the MD group were significantly decreased, and after ATM intervention, the level of occludin protein was significantly increased to the normal level. Moreover, the expressions of ZO-1 and claudin-1 also showed a recovery trend. This suggests that db/db mice have increased intestinal permeability, which allows the entry of gut microbes and toxins into the circulation to activate the inflammatory response, which is inhibited by ATM intervention.

The integrated analysis of WB and omics data demonstrates that ATM may alters the composition of intestinal microbiota, increases SCFAs levels. SCFAs, serving as an energy source for colonic epithelial cells, could influence the expression of tight junction proteins, enhance intestinal barrier function, activate the AMPK/mTOR pathway, and modulate the immune system, thereby alleviating inflammation state and improving metabolic disorders in diabetic mice.

### 4.7 Co-occurrence network analysis

Finally, we investigated the mechanism of ATM in the treatment of T2DM by integrating microbiome, metabolome, and inflammation data. In the co-occurrence network, *Prevotella* and *Helicobacter* were the dominant pathogens and positively related to proinflammatory cytokines, while *Lactobacillus* was negatively associated with proinflammatory cytokines. Intestinal dysbiosis increases intestinal permeability, which allows the intestinal microbiota and toxins to enter the blood circulation, causing an inflammatory response and leading to abnormal glucose metabolism (Mishra et al., 2023). *Lactobacillus* can inhibit the inflammatory response of the mouse intestinal tract through TLR4/MYD88/NF-κB axis (Li et al., 2020). Mild chronic inflammation is a key factor mediating the progression of DM (Donath and Shoelson, 2011). *Lactobacillus* was positively related to the propionate increase, suggesting that ATM may decrease the propionate level by increasing *Lactobacillus* abundance. In the co-occurrence network, LPS was positively correlated with *Prevotella*, *Helicobacter* and inflammatory factors, and negatively related to *Lactobacillus* and inflammatory factors. LPS is a key factor causing inflammatory responses and leading to many inflammatory diseases (Cao et al., 2019). The interaction between LPS and TLR4 activates intracellular signaling via MYD88 to activate p38 and NF-κB (Negishi et al., 2006). The elevation of LPS triggers the transcription of TNF-α, IL-1β and IL-6 through the phosphorylated subunit p65 (Gilmore, 2006). Subsequently, these cytokines can enhance TLR4-mediated pathway activation, coordinating the inflammatory reaction into a vicious cycle (Takeuchi and Akira, 2010). Through co-occurrence network analysis, we confirmed that inflammation and gut microbiota exerted vital function in ATM’s treatment of T2DM.

Taken together, after ATM intervention, there was a decrease in *Helicobacter* and *Prevotella*, leading to reduced LPS production. Additionally, the increase in *Lactobacillus* boosted the levels of SCFAs, which serve as a fuel source for colonic epithelial cells. Thus resulted in enhanced expression of occludin, strengthening the intestinal barrier, and simultaneously activated the AMPK/mTOR pathway. All these actions regulated the immune system, leading to the decreases in TNF-α, IL-1β, IL-6, NF-κB, and IL-17A, an increase in IL-10. Consequently, this alleviated the inflammatory state and improved metabolic disorders in diabetic mice.

## 5 Conclusion

The administration of 160 mg/kg ATM for 8 weeks obviously reduced FBG and lipid levels in db/db mice. This treatment improved IR, alleviated hepatic steatosis and pancreatic injury, and mitigated the inflammatory response. Further exploration of mechanisms revealed that ATM may modulate the composition of gut microbiota, increasing the abundance of *Lactobacillus*, which in turn elevates the levels of SCFAs. The elevation of SCFAs, especially propionic acid, may activate the AMPK/mTOR pathway, leading to the decreases in TNF-α, IL-1β, IL-6, NF-κB, and IL-17A, an increase in IL-10, thereby alleviating the inflammatory state and improving glucolipid metabolic disorder in T2DM. Taken together, the present study uncovered a novel mechanism of ATM to improve metabolic disorders in T2DM, laid a scientific foundation for the clinical application of ATM in reducing blood sugar, fat and anti-inflammation, and opened up new opportunities for the treatment of diabetes.

## Data Availability

The data presented in the study are deposited in the NCBI repository, accession number PRJNA1221318.

## Glossary

ATM: artemether
ATM-L: low-dose artemether group
ATM-H: high-dose artemether group
BCA: bicinchoninic acid assay
DM: diabetes mellitus
ELISA: enzyme-linked immunosorbent assay
ESI: electrospray ionization
FBG: fasting blood glucose
FINS: fasting insulin
GC-MS: gas chromatography-mass spectrometry
GO: Gene Ontology
H&E: hematoxylin-eosin
IPGTT: intraperitoneal glucose tolerance test
IPITT: intraperitoneal insulin tolerance test
IR: insulin resistance
KEGG: Kyoto Encyclopedia of Genes and Genomes
LPS: lipopolysaccharide
MD: model group
MET: metformin
NC: normal control group
OPLS-DA: orthogonal partial least squares-discriminant analysis
PCA: principal component analysis
PPI: protein-protein interactions
PVDF: polyvinylidene fluoride
RIPA: Radio-Immunoprecipitation Assay
SDS-PAGE: sodium dodecyl sulfate-polyacrylamide gel electrophoresis
SCFAs: short-chain fatty acids
16S rRNA: 16S ribosomal RNA
UHPLC-MS/MS: Ultra high performance liquid chromatography-mass spectrometry
ALT: Alanine aminotransferase
TC: Total Cholesterol
C-P: C-Peptide
GHbA1c: Glycated Hemoglobin A1c
TNF-α: Tumor Necrosis Factor-alpha
IL-1β: Interleukin-1 beta
IL-6: Interleukin-6
NF-*κ*B: Nuclear Factor kappa-light-chain-enhancer of activated B cells
IL-17A: Interleukin-17A
IL-10: Interleukin-10
IL-22: Interleukin-22
TGF-*β*1: Transforming Growth Factor-beta 1
TLR4: Toll-Like Receptor 4
AMPK: AMP-activated Protein Kinase
mTOR: Mechanistic Target of Rapamycin
ZO-1: Zonula Occludens-1
IKB*α*: Inhibitor of Nuclear Factor kappa-B alpha.
