# Supplementary material for: Exploring the glucose-lowering and anti-inflammatory immune mechanism of artemether by AMPK/mTOR pathway and microbiome based on multi-omics
Source: Front Pharmacol. 2025 Feb 19;16:1520439. doi: 10.3389/fphar.2025.1520439 (PMC11879814; doi:10.3389/fphar.2025.1520439)
Supplement: Supplementary file 1 [file DataSheet1.docx]

**Supplementary Tables and Figures**

**Table S1** Multivariate statistical analysis of liver metabonomics in db/db mice.

| Group |  | PCA, OPLS-DA score | | | Permutation test | |
| --- | --- | --- | --- | --- | --- | --- |
|  |  | R^2^X | R^2^Y | Q^2^ | R^2^ | Q^2^ |
| MD vs. NC | PCA | 0.515 | -- | -0.029 | -- | -- |
|  | OPLS-DA | 0.323 | 0.991 | 0.852 | (0, 0.8222) | (0, -1.06) |
| ATM-H vs. MD | PCA | 0.568 | -- | 0.552 | -- | -- |
|  | OPLS-DA | 0.439 | 0.991 | 0.791 | (0, 0.9565) | (0, -0.77) |

**Table S2** The 151 differential metabolites capable of callback under ATM intervention obtained by liver metabolomics analysis.

| No. | Rt  (min) | Adduct type | Metabolites | Caled m/z | Obsed m/z | Error (ppm) | Formula | CON vs MD | | | | MD vs ATM-H | | | |
| --- | --- | --- | --- | --- | --- | --- | --- | --- | --- | --- | --- | --- | --- | --- | --- |
|  |  |  |  |  |  |  |  | *P*. value | FC | VIP | Up/  Down | *P*. value | FC | VIP | Up/ Down |
| **1** | 3.763 | [M+H]^+^ | NAD | 664.114 | 664.117 | 4.502219 | C_21_H_28_N_7_O_14_P_2_^+^ | 0.002536 | 1.74 | 1.66 | Up | 0.011012 | 0.53 | 1.43 | Down |
| **2** | 8.65 | [M+Na]^+^ | Tuberostemonine | 398.24 | 398.2301 | 24.68422 | C_22_H_33_NO_4_ | 0.0017563 | 1.58 | 1.66 | Up | 0.000906 | 0.59 | 1.69 | Down |
| **3** | 4.506 | [M+H]^+^ | Homoharringtonine | 546.2654 | 546.2698 | 7.944792 | C_29_H_39_NO_9_ | 0.0061742 | 0.57 | 1.63 | Down | 0.006966 | 1.74 | 1.46 | Up |
| **4** | 10.162 | [M+H]^+^ | (6*S*,6a*S*,13*S*,13a*R*)-N-(benzo[d][1,3]dioxol-5-ylmethyl)-2,3,4,6,6a,7,8, 9,10,12,13,13a-dodecahydro-1H-6,13-methanodipyrido[1,2-a | 410.2393 | 410.2438 | 10.92034 | C_24_H_31_N_3_O_3_ | 0.008955 | 1.86 | 1.46 | Up | 4.65E-05 | 0.16 | 1.89 | Down |
| **5** | 5.216 | [M+Na]^+^ | Vincadifformine (1+) | 362.2061 | 362.2 | 16.78631 | C_21_H_27_N_2_O_2_+ | 0.0018374 | 0.60 | 1.73 | Down | 2.43E-05 | 3.91 | 1.94 | Up |
| **6** | 5.171 | [M-H]^-^ | Hippuric acid | 178.0516 | 178.0509 | 4.043787 | C_9_H_9_NO_3_ | 0.0404094 | 1.77 | 1.26 | Up | 0.013921 | 0.45 | 1.33 | Down |
| **7** | 10.312 | [M+H]^+^ | Dibutyl phthalate | 279.1586 | 279.1591 | 1.755272 | C_16_H_22_O_4_ | 2.323E-05 | 2.48 | 2.00 | Up | 0.000494 | 0.55 | 1.68 | Down |
| **8** | 8.804 | [M+H]^+^ | 2-methoxy-6-[(triphenylmethoxy)methyl]-2H-3,4,5,6-tetrahydropyran-3,4,5-triol | 437.1957 | 437.1959 | 0.411715 | C_26_H_28_O_6_ | 1.476E-05 | 4.02 | 2.02 | Up | 0.000228 | 0.38 | 1.81 | Down |
| **9** | 4.663 | [M-H]^-^ | Phenylacetic acid | 135.0471 | 135.0451 | 14.66177 | C_8_H_8_O_2_ | 0.0046444 | 1.87 | 1.58 | Up | 0.001396 | 0.43 | 1.72 | Down |
| **10** | 3.873 | [M+H]^+^ | 5,6-Dihydroxy-2-methylaminotetralin | 194.1155 | 194.1176 | 10.81819 | C_11_H_15_NO_2_ | 0.0027959 | 1.37 | 1.67 | Up | 0.000355 | 0.54 | 1.76 | Down |
| **11** | 8.961 | [M+H]^+^ | Benzophenone | 183.0802 | 183.0805 | 1.310899 | C_13_H_10_O | 6.184E-06 | 1.47 | 2.06 | Up | 2.57E-05 | 0.72 | 1.91 | Down |
| **12** | 7.642 | [M+H]^+^ | Capsaicin | 306.2116 | 306.2064 | 16.85138 | C_18_H_27_NO_3_ | 0.0015206 | 4.35 | 1.68 | Up | 0.004475 | 0.33 | 1.53 | Down |
| **13** | 1.258 | [M+Na]^+^ | Isosulochrin | 355.0705 | 355.0788 | 23.45958 | C_17_H_16_O_7_ | 0.021173 | 1.43 | 1.35 | Up | 0.040861 | 0.66 | 1.20 | Down |
| **14** | 5.953 | [M+Na]^+^ | Anisomycin | 288.1216 | 288.1206 | 3.470769 | C_14_H_19_NO_4_ | 0.0009335 | 0.39 | 1.70 | Down | 0.026098 | 2.06 | 1.25 | Up |
| **15** | 8.104 | [M+H]^+^ | Metoprolol acid | 268.1556 | 268.1543 | 5.108999 | C_14_H_21_NO_4_ | 0.0002045 | 1.649 | 1.85 | Up | 0.000518 | 0.69 | 1.73 | Down |
| **16** | 4.874 | [M-H]^-^ | Dimethachlor CGA369873 | 242.0508 | 242.0492 | 6.44497 | C_10_H_13_NO_4_S | 7.033E-06 | 5.58 | 2.08 | Up | 0.000856 | 0.39 | 1.71 | Down |
| **17** | 3.771 | [M+H]^+^ | Pterosin G | 235.128 | 235.1329 | 20.88181 | C_14_H_18_O_3_ | 0.0420764 | 0.62 | 1.26 | Down | 0.008185 | 2.98 | 1.46 | Up |
| **18** | 8.237 | [M+Na]^+^ | Eudesmin | 409.1646 | 409.1621 | 6.036727 | C_22_H_26_O_6_ | 5.777E-06 | 3.46 | 2.06 | Up | 0.000257 | 0.53 | 1.77 | Down |
| **19** | 8.778 | [M-H_2_O+H]^+^ | Niranthin | 415.212 | 415.211 | 2.504751 | C_24_H_32_O_7_ | 0.000255 | 1.97 | 1.85 | Up | 0.003561 | 0.63 | 1.50 | Down |
| **20** | 2.24 | [M+H]^+^ | Acetylcarnitine | 204.1223 | 204.123 | 3.184355 | C_9_H_17_NO_4_ | 0.0039836 | 3.65 | 1.63 | Up | 0.000563 | 0.18 | 1.74 | Down |
| **21** | 3.863 | [M+H]^+^ | Propionylcarnitine | 218.1366 | 218.1387 | 9.581061 | C_10_H_19_NO_4_ | 0.0204069 | 1.82 | 1.40 | Up | 0.001321 | 0.37 | 1.68 | Down |
| **22** | 11.211 | [M+H]^+^ | Docosahexaenoic acid | 329.2478 | 329.2475 | 1.093402 | C_22_H_32_O_2_ | 1.689E-06 | 2.15 | 2.11 | Up | 0.000312 | 0.78 | 1.76 | Down |
| **23** | 8.895 | [M+H]^+^ | Arachidonoylcarnitine | 448.3403 | 448.3421 | 3.947878 | C_27_H_45_NO_4_ | 0.0003064 | 2.02 | 1.87 | Up | 0.002299 | 0.57 | 1.64 | Down |
| **24** | 10.655 | [M+H-H_2_O]^+^ | 9-HOTrE | 277.2198 | 277.216 | 13.67165 | C_18_H_30_O_3_ | 0.0002969 | 2.68 | 1.83 | Up | 0.003104 | 0.48 | 1.55 | Down |
| **25** | 9.34 | [M+H]^+^ | Palmitoylcarnitine | 400.3428 | 400.3421 | 1.823441 | C_23_H_45_NO_4_ | 0.0025585 | 2.66 | 1.67 | Up | 0.000553 | 0.30 | 1.78 | Down |
| **26** | 8.912 | [M+H]^+^ | Linoleoylcarnitine | 424.3442 | 424.3421 | 5.113799 | C_25_H_45_NO_4_ | 0.0060916 | 1.81 | 1.52 | Up | 0.002507 | 0.48 | 1.64 | Down |
| **27** | 4.835 | [M+H]^+^ | Pivaloylcarnitine | 246.169 | 246.1705 | 6.133959 | C_12_H_23_NO_4_ | 0.0009758 | 2.31 | 1.77 | Up | 0.002598 | 0.37 | 1.62 | Down |
| **28** | 10.433 | [M+H]^+^ | LPE (18:1) | 480.3071 | 480.3085 | 2.914793 | C_23_H_46_NO_7_P | 0.0099171 | 1.82 | 1.48 | Up | 0.002223 | 0.45 | 1.57 | Down |
| **29** | 5.527 | [M+H]^+^ | L-Hexanoylcarnitine | 260.1869 | 260.1856 | 5.15017 | C_13_H_25_NO_4_ | 0.0398321 | 2.25 | 1.25 | Up | 0.025809 | 0.36 | 1.29 | Down |
| **30** | 8.558 | [M+CH_3_OH+H]^+^ | 3-Hydroxyhexadecanoylcarnitine | 416.3392 | 416.3376 | 3.939111 | C_23_H_45_NO_5_ | 0.0010112 | 3.01 | 1.72 | Up | 0.000687 | 0.30 | 1.76 | Down |
| **31** | 8.993 | [M+H-H_2_O]^+^ | -Icosa-8,11,14,17-tetraenoylcarnitine | 448.3396 | 448.3427 | 6.869745 | C_27_H_45_NO_4_ | 0.0006154 | 2.02 | 1.78 | Up | 0.000406 | 0.48 | 1.77 | Down |
| **32** | 3.959 | [M+H]^+^ | (2E)-Hexenedioylcarnitine | 288.1443 | 288.1447 | 1.561715 | C_13_H_21_NO_6_ | 1.208E-06 | 2.222 | 2.11 | Up | 0.000565 | 0.48 | 1.73 | Down |
| **33** | 9.345 | [M+H]^+^ | Oleoylcarnitine | 426.3549 | 426.3578 | 6.872162 | C_25_H_47_NO_4_ | 0.0004201 | 2.612 | 1.84 | Up | 0.001577 | 0.35 | 1.67 | Down |
| **34** | 6.049 | [M+CH_3_OH+H]^+^ | Heptanoylcarnitine | 274.2004 | 274.2018 | 5.105728 | C_14_H_27_NO_4_ | 0.0140023 | 3.602 | 1.42 | Up | 0.039292 | 0.26 | 1.20 | Down |
| **35** | 8.71 | [2M+H]^+^ | 3-Hydroxy-11Z-octadecenoylcarnitine | 442.3507 | 442.3532 | 5.78723 | C_25_H_47_NO_5_ | 0.0446659 | 2.25 | 1.21 | Up | 0.038916 | 0.33 | 1.24 | Down |
| **36** | 2.05 | [M+H]^+^ | delta-Valerobetaine | 160.1334 | 160.1332 | 1.311408 | C_8_H_17_NO_2_ | 0.02015 | 1.60 | 1.29 | Up | 0.000431 | 0.44 | 1.77 | Down |
| **37** | 3.625 | [M+CH_3_OH+H]^+^ | Succinylcarnitine | 262.1308 | 262.1291 | 6.523504 | C_11_H_19_NO_6_ | 0.0350383 | 4.45 | 1.25 | Up | 0.034964 | 0.20 | 1.19 | Down |
| **38** | 6.978 | [M+H]^+^ | Cortisol | 363.2156 | 363.2166 | 2.670583 | C_21_H_30_O_5_ | 0.0003101 | 15.47 | 1.87 | Up | 0.049023 | 0.72 | 1.23 | Down |
| **39** | 1.215 | [M+H]^+^ | 4-aminovaleric acid betaine | 160.1336 | 160.1332 | 2.497921 | C_8_H_17_NO_2_ | 0.018089 | 1.37 | 1.27 | Up | 0.000786 | 0.51 | 1.72 | Down |
| **40** | 3.776 | [M+H]^+^ | 3-Hydroxybutyrylcarnitine | 248.1475 | 248.1498 | 9.228297 | C_11_H_21_NO_5_ | 0.0304531 | 1.63 | 1.25 | Up | 0.003387 | 0.33 | 1.52 | Down |
| **41** | 8.125 | [M+H]^+^ | 3-Hydroxy-9-hexadecenoylcarnitine | 414.3225 | 414.322 | 1.32747 | C_23_H_43_NO_5_ | 0.0188492 | 1.82 | 1.36 | Up | 0.03175 | 0.41 | 1.27 | Down |
| **42** | 5.824 | [M+H-H_2_O]^+^ | Hept-4-enoylcarnitine | 272.1861 | 272.1862 | 0.440875 | C_14_H_25_NO_4_ | 0.0074293 | 3.45 | 1.49 | Up | 0.001035 | 0.10 | 1.67 | Down |
| **43** | 10.654 | [M-H_2_O-H]^-^ | 4,8,12,15-Octadecatetraenoic acid | 275.202 | 275.2011 | 3.233999 | C_18_H_28_O_2_ | 0.0018433 | 2.31 | 1.70 | Up | 0.030132 | 0.60 | 1.19 | Down |
| **44** | 6.474 | [M+H]^+^ | (2*E*,6*E,*8*E*)-Decatrienoylcarnitine | 310.2019 | 310.2018 | 0.096711 | C_17_H_27_NO_4_ | 0.001916 | 4.79 | 1.68 | Up | 0.006797 | 0.29 | 1.51 | Down |
| **45** | 8.751 | [M+H-H_2_O]^+^ | Trans-Hexadec-2-Enoyl Carnitine | 398.3237 | 398.327 | 8.28465 | C_23_H_43_NO_4_ | 0.0420806 | 1.95 | 1.25 | Up | 0.028699 | 0.43 | 1.30 | Down |
| **46** | 4.659 | [2M+H]^+^ | Oct-5-enedioylcarnitine | 316.1804 | 316.176 | 13.88467 | C_15_H_25_NO_6_ | 0.0347243 | 2.11 | 1.15 | Up | 0.006037 | 0.35 | 1.50 | Down |
| **47** | 8.779 | [M+ACN+H]^+^ | 7b,9-Dihydroxy-3-(hydroxymethyl)-1,1,6,8-tetramethyl-5-oxo-1,1a,1b,4,4a, 5,7a,7b,8,9-decahydro-9ah-cyclopropa[3,4]benzo[1,2-e]azulen-9a-yl acetate | 432.238 | 432.238 | 0.069406 | C_22_H_30_O_6_ | 1.376E-05 | 2.41 | 2.02 | Up | 0.000734 | 0.58 | 1.67 | Down |
| **48** | 7.344 | [M-H_2_O-H]^-^ | 11b,21-Dihydroxy-3,20-oxo-5b-pregnan-18-al | 363.2165 | 363.2172 | 1.844627 | C_21_H_32_O_5_ | 0.0002151 | 3.44 | 1.91 | Up | 0.042517 | 0.56 | 1.13 | Down |
| **49** | 10.545 | [M-H]^-^ | Hexadecatrienoic acid | 249.1849 | 249.186 | 4.494635 | C_16_H_26_O_2_ | 0.0002786 | 5.51 | 1.86 | Up | 0.020476 | 0.50 | 1.25 | Down |
| **50** | 5.301 | [M+NH_4_]^+^ | 2-Hexenoylcarnitine | 258.1693 | 258.1705 | 4.609356 | C_13_H_23_NO_4_ | 0.0160747 | 2.79 | 1.41 | Up | 0.001248 | 0.13 | 1.63 | Down |
| **51** | 9.64 | [M+H]^+^ | 5alpha-Androstane-3beta,7alpha,17beta-triol | 309.2435 | 309.2424 | 3.363057 | C_19_H_32_O_3_ | 0.0105092 | 4.68 | 1.46 | Up | 0.028725 | 0.33 | 1.23 | Down |
| **52** | 11.067 | [M+H]^+^ | alpha-Eleostearic acid | 279.2314 | 279.2318 | 1.754814 | C_18_H_30_O_2_ | 0.0004257 | 2.90 | 1.81 | Up | 0.036805 | 0.65 | 1.12 | Down |
| **53** | 4.275 | [M-H]^-^ | 3-Hydroxyisovaleric acid | 117.056 | 117.0557 | 2.562882 | C_5_H_10_O_3_ | 0.0010651 | 2.57 | 1.70 | Up | 0.000126 | 0.35 | 1.83 | Down |
| **54** | 9.426 | [M+Na]^+^ | (1*S*,2*R*,4a*R*,8a*R*)-1-Acetoxy-7-isopropylidene-1,4a-dimethyl-6-oxodecahydro-2-naphthalenyl 2,3-dimethyl-2-oxiranecarboxylate | 415.2107 | 415.2091 | 3.877564 | C_22_H_32_O_6_ | 0.0014227 | 2.19 | 1.69 | Up | 0.038038 | 0.65 | 1.18 | Down |
| **55** | 7.817 | [M+CH_3_OH+H]^+^ | 3-Hydroxyhexadecadienoylcarnitine | 412.3052 | 412.3063 | 2.716427 | C_23_H_41_NO_5_ | 0.0226233 | 2.68 | 1.34 | Up | 0.022067 | 0.34 | 1.32 | Down |
| **56** | 5.452 | [M+H]^+^ | thujopsenone | 219.1726 | 219.1744 | 7.938884 | C_15_H_22_O | 8.638E-05 | 3.86 | 1.93 | Up | 7.17E-05 | 0.21 | 1.88 | Down |
| **57** | 9.01 | [M+H-H_2_O]^+^ | 5(*S*)-HETE | 303.2286 | 303.231 | 8.046671 | C_20_H_32_O_3_ | 0.0324344 | 0.78 | 1.33 | Down | 0.030965 | 1.60 | 1.20 | Up |
| **58** | 10.055 | [M-H]^-^ | Hexadeca-4,7,10,13-tetraenoate | 247.1729 | 247.1704 | 10.31677 | C_16_H_24_O_2_ | 0.000233 | 2.79 | 1.87 | Up | 0.006015 | 0.55 | 1.46 | Down |
| **59** | 7.345 | [M+H]^+^ | Tetrahydrocortisone | 365.2345 | 365.2323 | 6.105704 | C_21_H_32_O_5_ | 2.176E-05 | 2.60 | 2.03 | Up | 0.003126 | 0.45 | 1.53 | Down |
| **60** | 10.86 | [M-H]^-^ | 7*Z*,10*Z*-Hexadecadienoic acid | 251.2026 | 251.2011 | 6.01112 | C_16_H_28_O_2_ | 0.0091997 | 4.64 | 1.48 | Up | 0.038104 | 0.51 | 1.14 | Down |
| **61** | 4.486 | [M-H]^-^ | 2-Ethyl-2-hydroxybutyric acid | 131.0705 | 131.0713 | 6.179842 | C_6_H_12_O_3_ | 0.0015263 | 2.40 | 1.69 | Up | 0.001175 | 0.45 | 1.66 | Down |
| **62** | 8.449 | [M-H]^-^ | TetraHCA | 465.3233 | 465.3216 | 3.674878 | C_27_H_46_O_6_ | 0.0001009 | 0.32 | 1.92 | Down | 0.007672 | 1.58 | 1.49 | Up |
| **63** | 8.359 | [M+CH^3^OH+H]^+^ | 3, 5-Tetradecadiencarnitine | 368.2729 | 368.2801 | 19.46888 | C_21_H_37_NO_4_ | 0.0003053 | 4.11 | 1.84 | Up | 0.010559 | 0.38 | 1.47 | Down |
| **64** | 1.825 | [M+Na]^+^ | Utilin | 839.3189 | 839.3096 | 10.98522 | C_41_H_52_O_17_ | 0.0341356 | 0.43 | 1.32 | Down | 0.008549 | 5.24 | 1.47 | Up |
| **65** | 6.108 | [M-H_2_O+H]^+^ | 8-[1-(acetyloxy)-4-oxo-5-[(3*E*)-2-oxopent-3-en-1-yl]cyclopent-2-en-1-yl]octanoic acid | 347.1786 | 347.185 | 18.54919 | C_20_H_28_O_6_ | 0.0001296 | 5.81 | 1.90 | Up | 0.038543 | 0.53 | 1.13 | Down |
| **66** | 4.486 | [M+H]^+^ | Ranaconitine | 601.3133 | 601.312 | 2.228461 | C_32_H_44_N_2_O_9_ | 0.0397706 | 0.61 | 1.32 | Down | 0.005236 | 2.52 | 1.50 | Up |
| **67** | 7.488 | [M+H]^+^ | Fuziline | 454.2788 | 454.2799 | 2.355376 | C_24_H_39_NO_7_ | 0.0116031 | 0.49 | 1.45 | Down | 0.00836 | 2.20 | 1.44 | Up |
| **68** | 8.78 | [M+Na]^+^ | Neoline | 460.2721 | 460.2669 | 11.34125 | C_24_H_39_NO_6_ | 3.735E-08 | 12.55 | 2.21 | Up | 4.32E-06 | 0.29 | 1.98 | Down |
| **69** | 11.011 | [M+H+CH_3_CN]^+^ | 22-Hydoxy-2-hopen-1-one | 482.4053 | 482.4 | 11.06965 | C_30_H_48_O_2_ | 0.0013657 | 2.71 | 1.69 | Up | 0.027903 | 0.59 | 1.20 | Down |
| **70** | 8.784 | [M+Na]^+^ | Methylprednisolone | 397.2012 | 397.1985 | 6.747256 | C_22_H_30_O_5_ | 0.000359 | 2.14 | 1.85 | Up | 0.004078 | 0.61 | 1.48 | Down |
| **71** | 7.83 | [M+Na]^+^ | Prostaglandin E1 | 377.2248 | 377.2298 | 13.28103 | C_20_H_34_O_5_ | 0.000157 | 3.56 | 1.89 | Up | 0.012453 | 0.56 | 1.41 | Down |
| **72** | 4.631 | [M+Na]^+^ | Foliandrin | 599.3117 | 599.319 | 12.31398 | C_32_H_48_O_9_ | 0.0427165 | 0.61 | 1.28 | Down | 0.007769 | 3.82 | 1.47 | Up |
| **73** | 3.775 | [M+H]^+^ | Adenosine | 268.1049 | 268.104 | 3.431505 | C_10_H_13_N_5_O_4_ | 0.0050039 | 2.32 | 1.57 | Up | 0.00348 | 0.36 | 1.58 | Down |
| **74** | 3.818 | [M+H]^+^ | Inosine | 269.0897 | 269.0881 | 6.131821 | C_10_H_12_N_4_O_5_ | 0.0125359 | 1.23 | 1.39 | Up | 0.001416 | 0.66 | 1.64 | Down |
| **75** | 2.611 | [M+H]^+^ | dGMP | 348.069 | 348.0704 | 4.022175 | C_10_H_14_N_5_O_7_P | 0.0488223 | 2.21 | 1.18 | Up | 0.007758 | 0.18 | 1.45 | Down |
| **76** | 3.74 | [M+H]^+^ | Uridine | 245.0772 | 245.0768 | 1.550534 | C_9_H_12_N_2_O_6_ | 0.0164919 | 2.07 | 1.40 | Up | 0.013424 | 0.45 | 1.37 | Down |
| **77** | 7.212 | [2M+H]^+^ | 2-Methylthio-N6-(delta2-isopentenyl) adenosine | 382.1571 | 382.1544 | 7.169878 | C_16_H_23_N_5_O4_S_ | 0.0356589 | 0.634312 | 1.31 | Down | 0.028306 | 2.59 | 1.29 | Up |
| **78** | 4.644 | [M+H-H2O]^+^ | FMN | 439.1016 | 439.102 | 0.91095 | C_17_H_21_N_4_O_9_P | 0.002654 | 1.60 | 1.63 | Up | 0.000491 | 0.52 | 1.76 | Down |
| **79** | 4.632 | [M+H]^+^ | FAD | 786.165 | 786.1644 | 0.852239 | C_27_H_33_N_9_O_15_P_2_ | 0.0083071 | 1.37 | 1.47 | Up | 0.005104 | 0.70 | 1.51 | Down |
| **80** | 1.829 | [M+H]^+^ | Adenosine 3',5'-diphosphate | 428.0352 | 428.0367 | 3.410923 | C_10_H_15_N_5_O_10_P_2_ | 0.0456542 | 2.45 | 1.24 | Up | 0.018284 | 0.26 | 1.36 | Down |
| **81** | 1.87 | [M+H]^+^ | UMP | 325.0464 | 325.0432 | 9.783316 | C_9_H_13_N_2_O_9_P | 0.0361029 | 2.67 | 1.30 | Up | 0.014229 | 0.16 | 1.35 | Down |
| **82** | 2.879 | [M-H]^-^ | N-Acetylglutamic acid | 188.0557 | 188.0564 | 3.934989 | C_7_H_11_NO_5_ | 0.0156181 | 2.25 | 1.38 | Up | 0.032197 | 0.35 | 1.27 | Down |
| **83** | 4.766 | [M-H]^-^ | N-Acetylmethionine | 190.0564 | 190.0543 | 10.99686 | C_7_H_13_NO_3_S | 0.007199 | 1.68 | 1.51 | Up | 0.037611 | 0.68 | 1.20 | Down |
| **84** | 2.044 | [M-H]^-^ | 2-Hydroxyglutaric acid | 147.0282 | 147.0299 | 11.6983 | C_5_H_8_O_5_ | 0.0496029 | 1.90 | 1.21 | Up | 0.040094 | 0.51 | 1.21 | Down |
| **85** | 4.97 | [M+H]^+^ | Phe-Pro | 263.1404 | 263.139 | 5.206374 | C_14_H_18_N_2_O_3_ | 0.0043191 | 0.61 | 1.68 | Down | 0.009052 | 3.53 | 1.42 | Up |
| **86** | 2.082 | [M+H]^+^ | gamma-Guanidinobutyric acid | 146.0916 | 146.0924 | 5.133738 | C_5_H_11_N_3_O_2_ | 0.0005261 | 3.47 | 1.80 | Up | 0.005138 | 0.42 | 1.56 | Down |
| **87** | 1.829 | [M+H]^+^ | Pipecolic acid | 130.0845 | 130.0863 | 13.37574 | C_6_H_11_NO_2_ | 2.775E-06 | 3.54 | 2.09 | Up | 9.36E-05 | 0.49 | 1.80 | Down |
| **88** | 5.895 | [M+H]^+^ | N-acetyltryptophan | 247.11 | 247.1077 | 9.348151 | C_13_H_14_N_2_O_3_ | 0.0003094 | 5.16 | 1.87 | Up | 0.004509 | 0.35 | 1.52 | Down |
| **89** | 13.273 | [M+H]^+^ | 3-Amino-2,2-dimethylpropanoic acid | 118.0853 | 118.0863 | 7.790915 | C_5_H_11_NO_2_ | 0.0016156 | 1.55 | 1.69 | Up | 0.03662 | 0.84 | 1.14 | Down |
| **90** | 5.333 | [M+H]^+^ | N-Acetylisoleucine | 174.112 | 174.1125 | 2.699405 | C_8_H_15_NO_3_ | 0.0002346 | 3.05 | 1.86 | Up | 0.000531 | 0.39 | 1.72 | Down |
| **91** | 5.451 | [M-H]^-^ | Acetylleucine | 172.099 | 172.0979 | 5.984965 | C_8_H_15_NO_3_ | 0.0002201 | 4.48 | 1.89 | Up | 0.005751 | 0.43 | 1.47 | Down |
| **92** | 0.891 | [M+H]^+^ | Ornithine | 133.0944 | 133.0972 | 20.96213 | C_5_H_12_N_2_O_2_ | 0.0458843 | 0.68 | 1.26 | Down | 0.001422 | 2.71 | 1.69 | Up |
| **93** | 3.794 | [M-H]^-^ | *D*-4'-Phosphopantothenate | 298.0673 | 298.0692 | 6.34081 | C_9_H_18_NO_8_P | 0.0483627 | 0.57 | 1.23 | Down | 0.04897 | 1.78 | 1.15 | Up |
| **94** | 4.457 | [M+H]^+^ | *L*-beta-Homoleucine | 146.118 | 146.1176 | 3.14815 | C_7_H_15_NO_2_ | 0.0254625 | 0.75 | 1.33 | Down | 0.003739 | 1.81 | 1.53 | Up |
| **95** | 4.043 | [M+H]^+^ | Methyl DL-methionate | 164.0731 | 164.074 | 5.058694 | C_6_H_13_NO_2_S | 0.0080539 | 0.66 | 1.46 | Down | 0.048436 | 1.43 | 1.09 | Up |
| **96** | 4.898 | [M-H]^-^ | N-Lactoylvaline | 188.0923 | 188.0923 | 0 | C_8_H_15_NO_4_ | 0.0073043 | 2.09 | 1.51 | Up | 0.043798 | 0.59 | 1.17 | Down |
| **97** | 1.979 | [M+H]^+^ | Leu-His | 269.1629 | 269.1608 | 7.802029 | C_12_H_20_N_4_O_3_ | 0.0247935 | 0.45 | 1.44 | Down | 0.011386 | 6.96 | 1.40 | Up |
| **98** | 5.887 | [M-H]^-^ | N-Acetyltryptophan | 245.0939 | 245.0931 | 3.060061 | C_13_H_14_N_2_O_3_ | 0.0003094 | 5.16 | 1.87 | Up | 0.004509 | 0.35 | 1.52 | Down |
| **99** | 8.572 | [M+H]^+^ | Tris (2-chloroisopropyl) phosphate | 327.0095 | 327.0081 | 4.28124 | C_9_H_18_Cl_3_O_4_P | 0.000242 | 2.14 | 1.88 | Up | 0.001985 | 0.60 | 1.57 | Down |
| **100** | 5.16 | [M-H]^-^ | 3-(3-Sulfooxyphenyl) propanoic acid | 245.012 | 245.0125 | 2.081526 | C_9_H_10_O_6_S | 0.0375461 | 2.35 | 1.28 | Up | 0.01873 | 0.38 | 1.26 | Down |
| **101** | 0.924 | [M+H]^+^ | Cyclo-prolylglycine | 155.0772 | 155.0815 | 27.34047 | C_7_H_10_N_2_O_2_ | 0.0220891 | 0.61 | 1.41 | Down | 0.008028 | 2.73 | 1.45 | Up |
| **102** | 4.765 | [M+Na]^+^ | 6-Oxopiperidine-2-carboxylic acid | 144.0643 | 144.0661 | 12.07779 | C_6_H_9_NO_3_ | 0.0050829 | 1.67 | 1.57 | Up | 0.027399 | 0.67 | 1.27 | Down |
| **103** | 4.348 | [M+H]^+^ | Val-Gly-Val | 274.1729 | 274.1761 | 11.7078 | C_12_H_23_N_3_O_4_ | 0.0423138 | 0.64 | 1.20 | Down | 0.003107 | 3.16 | 1.55 | Up |
| **104** | 4.829 | [M+H]^+^ | 4-[(2,4-Dihydroxy-3,3-dimethylbutanoyl) amino] butanoic acid | 234.1349 | 234.1336 | 5.381543 | C_10_H_19_NO_5_ | 0.0003311 | 1.93 | 1.85 | Up | 0.002506 | 0.57 | 1.61 | Down |
| **105** | 8.032 | [M+H]^+^ | IRB_p_329.1858_19.4 | 329.1856 | 329.186 | 1.093607 | C_19_H_24_N_2_O_3_ | 0.0198485 | 2.33 | 1.31 | Up | 0.00138 | 0.19 | 1.69 | Down |
| **106** | 1.196 | [M-H]^-^ | N-Acetyltaurine | 166.0155 | 166.0174 | 11.50482 | C_4_H_9_NO_4_S | 8.124E-05 | 3.44 | 1.95 | Up | 0.002944 | 0.48 | 1.63 | Down |
| **107** | 4.534 | [M+H]^+^ | N-((*S*)-1,2,3-trimethoxy-10-(((*S*)-3-methyl-1-oxo-1-(4-(2,3,4-trimethoxybenzyl) piperazin-1-yl)butan-2-yl) amino)-9-oxo-5,6,7,9-tetrahydrobenzo [a]heptalen-7-yl) acetamide | 733.3774 | 733.3807 | 4.499709 | C_40_H_52_N_4_O_9_ | 0.0013662 | 0.44 | 1.75 | Down | 0.003696 | 5.51 | 1.53 | Up |
| **108** | 1.156 | [M+H]^+^ | Carnitine | 162.1125 | 162.1125 | 0.123371 | C_7_H_15_NO_3_ | 0.0005594 | 1.53 | 1.77 | Up | 0.002102 | 0.65 | 1.57 | Down |
| **109** | 1.205 | [M+NH_4_]^+^ | Trehalose | 360.15 | 360.15 | 0.166597 | C_12_H_22_O_11_ | 0.0051962 | 1.60 | 1.57 | Up | 0.016472 | 0.69 | 1.29 | Down |
| **110** | 9.895 | [M+H]^+^ | 3,5-Di-tert-butyl-4-hydroxybenzaldehyde | 235.1697 | 235.1693 | 1.743425 | C_15_H_22_O_2_ | 4.136E-05 | 1.84 | 2.02 | Up | 0.000149 | 0.61 | 1.78 | Down |
| **111** | 9.556 | [M-H]^-^ | Mannose | 179.055 | 179.0561 | 6.534264 | C_6_H_12_O_6_ | 0.001201 | 2.81 | 1.717 | Up | 0.01256 | 0.50 | 1.30 | Down |
| **112** | 4.363 | [M-H]^-^ | 1-Hydroxy-2-butanone | 87.04469 | 87.0446 | 1.033953 | C_4_H_8_O_2_ | 0.0009408 | 2.30 | 1.767 | Up | 0.001064 | 0.41 | 1.76 | Down |
| **113** | 10.891 | [M-H]^-^ | Humulone | 361.1992 | 361.202 | 7.77958 | C_21_H_30_O_5_ | 0.0295266 | 3.78 | 1.27 | Up | 0.027136 | 0.25 | 1.26 | Down |
| **114** | 10.593 | [M-H]^-^ | Cohumulone | 347.1837 | 347.1864 | 8.007225 | C_20_H_28_O_5_ | 0.0129887 | 2.87 | 1.42 | Up | 0.005415 | 0.27 | 1.53 | Down |
| **115** | 3.768 | [M+H]^+^ | S-Adenosylhomocysteine | 385.1306 | 385.1289 | 4.414107 | C_14_H_20_N_6_O5_S_ | 0.0025756 | 1.82 | 1.65 | Up | 0.002948 | 0.48 | 1.59 | Down |
| **116** | 3.825 | [M+H]^+^ | Hypoxanthine | 137.044 | 137.0458 | 12.69649 | C_5_H_4_N_4_O | 0.0042944 | 1.28 | 1.57 | Up | 0.000573 | 0.62 | 1.72 | Down |
| **117** | 13.278 | [M+H]^+^ | Methenamine | 141.1109 | 141.1135 | 18.56662 | C_6_H_12_N_4_ | 9.827E-05 | 1.53 | 1.90 | Up | 0.000395 | 0.77 | 1.71 | Down |
| **118** | 2.956 | [M-H]^-^ | Uric acid | 167.0211 | 167.021 | 0.419109 | C_5_H_4_N_4_O_3_ | 0.0001387 | 2.85 | 1.95 | Up | 0.021174 | 0.51 | 1.35 | Down |
| **119** | 10.315 | [M+H]^+^ | Phthalic anhydride | 149.0214 | 149.0233 | 12.74969 | C_8_H_4_O_3_ | 1.005E-05 | 2.66 | 2.05 | Up | 0.000736 | 0.56 | 1.64 | Down |
| **120** | 3.716 | [M-H]^-^ | UDP-glucuronate | 579.0242 | 579.027 | 4.852969 | C_15_H_22_N_2_O_18_P_2_ | 0.008933 | 12.44 | 1.49 | Up | 0.013969 | 0.13 | 1.32 | Down |
| **121** | 8.093 | [M+H]^+^ | 2,2,6,6-Tetramethylpiperidin-1-ol | 158.1536 | 158.154 | 2.149804 | C_9_H_19_NO | 0.0008744 | 1.41 | 1.76 | Up | 0.001531 | 0.76 | 1.67 | Down |
| **122** | 0.921 | [M+H]^+^ | 2-(hydroxymethyl) quinazolin-4(3h)-one | 177.0608 | 177.0659 | 28.68988 | C_9_H_8_N_2_O_2_ | 0.0238536 | 0.57 | 1.33 | Down | 0.023593 | 2.41 | 1.26 | Up |
| **123** | 8.782 | [M+H]^+^ | 5-O-Methylvisammioside | 453.1668 | 453.1755 | 19.26406 | C_22_H_28_O_10_ | 6.773E-06 | 4.80 | 2.05 | Up | 0.000488 | 0.44 | 1.73 | Down |
| **124** | 4.765 | [M+H]^+^ | Oxyquinoline | 146.0615 | 146.0601 | 9.448167 | C_9_H_7_NO | 0.0009408 | 1.71 | 1.77 | Up | 0.016666 | 0.68 | 1.33 | Down |
| **125** | 5.659 | [M-H]^-^ | PharmaGSID_47333 | 381.2291 | 381.2296 | 1.259084 | C_22_H_30_N_4_O_2_ | 0.002885 | 2.89 | 1.65 | Up | 0.019927 | 0.49 | 1.30 | Down |
| **126** | 1.165 | [M+H-H_2_O]^+^ | 1,6-Anhydro-beta-glucopyranose | 145.0493 | 145.047 | 15.6501 | C_6_H_10_O_5_ | 0.0227523 | 1.51 | 1.38 | Up | 0.003001 | 0.64 | 1.62 | Down |
| **127** | 3.819 | [M+Na]^+^ | Protostemotinine | 438.19 | 438.1887 | 2.989579 | C_23_H_29_NO_6_ | 0.0005731 | 0.30 | 1.82 | Down | 0.039313 | 1.75 | 1.20 | Up |
| **128** | 5.173 | [M-H]^-^ | 1,5,6,7-Tetrahydro-4H-indol-4-one | 134.06 | 134.0611 | 8.205212 | C_8_H_9_NO | 0.0260692 | 1.82 | 1.35 | Up | 0.01 | 0.49 | 1.37 | Down |
| **129** | 3.289 | [M+H]^+^ | Imidacloprid-urea | 212.0531 | 212.0585 | 25.55899 | C_9_H_10_ClN_3_O | 0.0010509 | 3.37 | 1.74 | Up | 0.01 | 0.39 | 1.49 | Down |
| **130** | 4.208 | [M+Na]^+^ | N-(2-(2-(2-hydroxy-5-oxo-4,5-dihydro-3H-benzo[e][1,4]diazepin-3-yl)acetamido)ethyl)-1-methyl-1H-indole-2-carboxamide | 456.161 | 456.1642 | 6.971174 | C_23_H_23_N_5_O_4_ | 0.0067584 | 4.68 | 1.52 | Up | 0.016522 | 0.28 | 1.34 | Down |
| **131** | 4.05 | [M+Na]^+^ | 2-methoxyethyl 5-hydroxy-2-methyl-1-benzylbenzo[g] indole-3-carboxylate | 412.1503 | 412.1519 | 3.930589 | C_24_H_23_NO_4_ | 0.020341 | 2.60 | 1.33 | Up | 0.029563 | 0.40 | 1.29 | Down |
| **132** | 4.873 | [M+H]^+^ | indol-2-one | 132.0451 | 132.0444 | 5.301248 | C_8_H_5_NO | 0.00383 | 3.04 | 1.61 | Up | 0.000635 | 0.19 | 1.71 | Down |
| **133** | 5.782 | [M+Na]^+^ | 3-Hydroxycarbofuran | 260.087 | 260.0893 | 8.996909 | C_12_H_15_NO_4_ | 5.918E-05 | 2.17 | 1.97 | Up | 0.034953 | 0.64 | 1.20 | Down |
| **134** | 5.004 | [M-H]^-^ | Stachybotramide | 428.2497 | 428.2442 | 12.67968 | C_25_H_35_NO_5_ | 0.001668 | 0.38 | 1.74 | Down | 0.018892 | 2.33 | 1.36 | Up |
| **135** | 8.239 | [M+Na]^+^ | N-(2-(((1*S*,9a*R*)-octahydro-1H-quinolizin-1-yl)methyl)-3-oxo-1,2,3,4-tetrahydrobenzo[4,5] imidazo[1,2-a]pyrazin-8-yl) propionamide | 432.2376 | 432.237 | 1.203044 | C_23_H_31_N_5_O_2_ | 1.415E-06 | 4.29 | 2.125 | Up | 6.34E-05 | 0.47 | 1.86 | Down |
| **136** | 6.222 | [M-H_2_O+H]^+^ | Loliolide | 179.1043 | 179.105 | 4.019988 | C_11_H_16_O_3_ | 6.07E-05 | 0.37 | 2.02 | Down | 0.019821 | 2.19 | 1.36 | Up |
| **137** | 6.003 | [M+Na]^+^ | N-(2-chlorophenyl)-2-[4-(N-methylcarbamoyl)-2-oxohydroquinolyl]acetamide | 392.0811 | 392.0772 | 9.972526 | C_19_H_16_ClN_3_O_3_ | 0.0242545 | 43.14 | 1.31 | Up | 0.026777 | 0.04 | 1.16 | Down |
| **138** | 4.455 | [M+Na]^+^ | 3-HYDROXY-4-(SUCCIN-2-YL)-CARYOLANE delta-LACTONE | 343.1905 | 343.1879 | 7.45947 | C_19_H_28_O_4_ | 0.0337016 | 0.41 | 1.33 | Down | 0.02508 | 3.007 | 1.33 | Up |
| **139** | 2.512 | [M-H]^-^ | N-Methyltryptamine | 173.1062 | 173.1084 | 12.99764 | C_11_H_14_N_2_ | 0.0139838 | 0.38 | 1.52 | Down | 0.006244 | 9.497 | 1.47 | Up |
| **140** | 4.668 | [M-H]^-^ | Hydroxyphenyllactic acid | 181.0506 | 181.0506 | 0.331399 | C_9_H_10_O_4_ | 0.0026038 | 1.68 | 1.65 | Up | 0.000108 | 0.427 | 1.89 | Down |
| **141** | 1.261 | [M+H]^+^ | Epigallocatechin | 307.0866 | 307.0812 | 17.78031 | C_15_H_14_O_7_ | 0.0244346 | 1.49 | 1.33 | Up | 0.018363 | 0.57 | 1.36 | Down |
| **142** | 5.777 | [M-H]^-^ | 3-Phenyllactic acid | 165.0557 | 165.0557 | 0.484685 | C_9_H_10_O_3_ | 0.0374237 | 3.62 | 1.24 | Up | 0.03298 | 0.26 | 1.25 | Down |
| **143** | 5.297 | [M-H]^-^ | Wogonoside | 459.0962 | 459.0933 | 6.186106 | C_22_H_20_O_11_ | 0.0102081 | 2.59 | 1.47 | Up | 0.004368 | 0.068 | 1.47 | Down |
| **144** | 5.245 | [M+H]^+^ | Daidzin | 417.1158 | 417.118 | 5.274287 | C_21_H_20_O_9_ | 0.0432755 | 8.95 | 1.18 | Up | 0.036422 | 0.08 | 1.13 | Down |
| **145** | 5.26 | [M-H]^-^ | Chrysin-7-O-glucuronide | 429.0819 | 429.0827 | 2.050887 | C_21_H_18_O_10_ | 0.0366407 | 2.55 | 1.24 | Up | 0.007849 | 0.22 | 1.43 | Down |
| **146** | 8.24 | [M+H]^+^ | 6,7-dimethyl-4-(((4-oxo-3-phenyl-4H-chromen-7-yl)oxy)methyl)-2H-chromen-2-one | 425.1346 | 425.1384 | 8.98531 | C_27_H_20_O_5_ | 2.156E-05 | 3.25 | 1.99 | Up | 0.001905 | 0.63 | 1.57 | Down |
| **147** | 4.116 | [M+H-H_2_O]^+^ | Cinnamic acid | 131.0494 | 131.049 | 2.899679 | C_9_H_8_O_2_ | 0.0008771 | 0.30 | 1.72 | Down | 0.001523 | 1.58 | 1.67 | Up |
| **148** | 4.99 | [M]^+^ | Caffeoylcholine | 266.137 | 266.1381 | 4.245916 | C_14_H_20_NO_4_ | 0.0010772 | 1.91 | 1.76 | Up | 7.27E-05 | 0.31 | 1.85 | Down |
| **149** | 5.475 | [M+Na]^+^ | Licochalcone A | 361.1477 | 361.141 | 18.52462 | C_21_H_22_O_4_ | 0.0086265 | 8.12 | 1.47 | Up | 0.01601 | 0.20 | 1.34 | Down |
| **150** | 5.567 | [M-H]^-^ | Quercetin | 301.0373 | 301.0353 | 6.477645 | C_15_H_10_O_7_ | 0.0332887 | 2.03 | 1.29 | Up | 0.047264 | 0.47 | 1.19 | Down |
| **151** | 3.903 | [M-H]^-^ | 2',6'-Dihydroxy-4-methoxychalcone-4'-O-neohesperid | 593.1884 | 593.1875 | 1.550943 | C_28_H_34_O_14_ | 0.0437341 | 0.44 | 1.23 | Down | 0.016405 | 1.98 | 1.29 | Up |


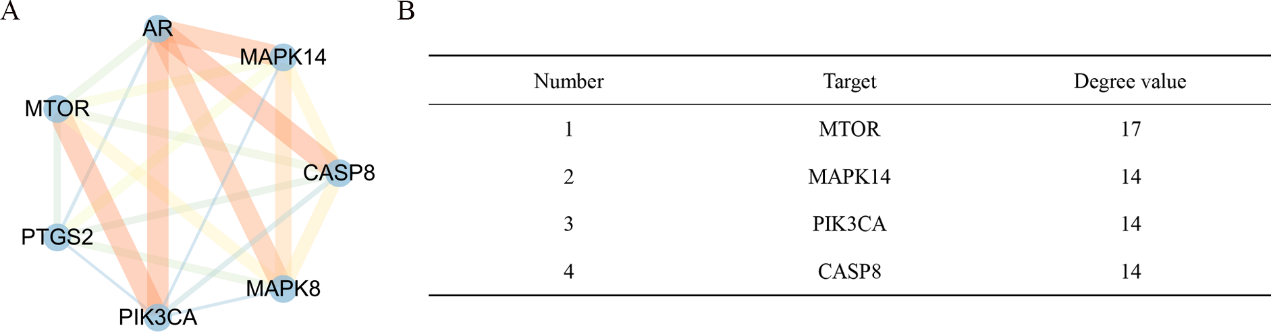


**Fig. S1.** Network pharmacological analysis. **(A)** Core targets enriched by network pharmacology. **(B)** The degree values of the TOP 4 enriched core targets.


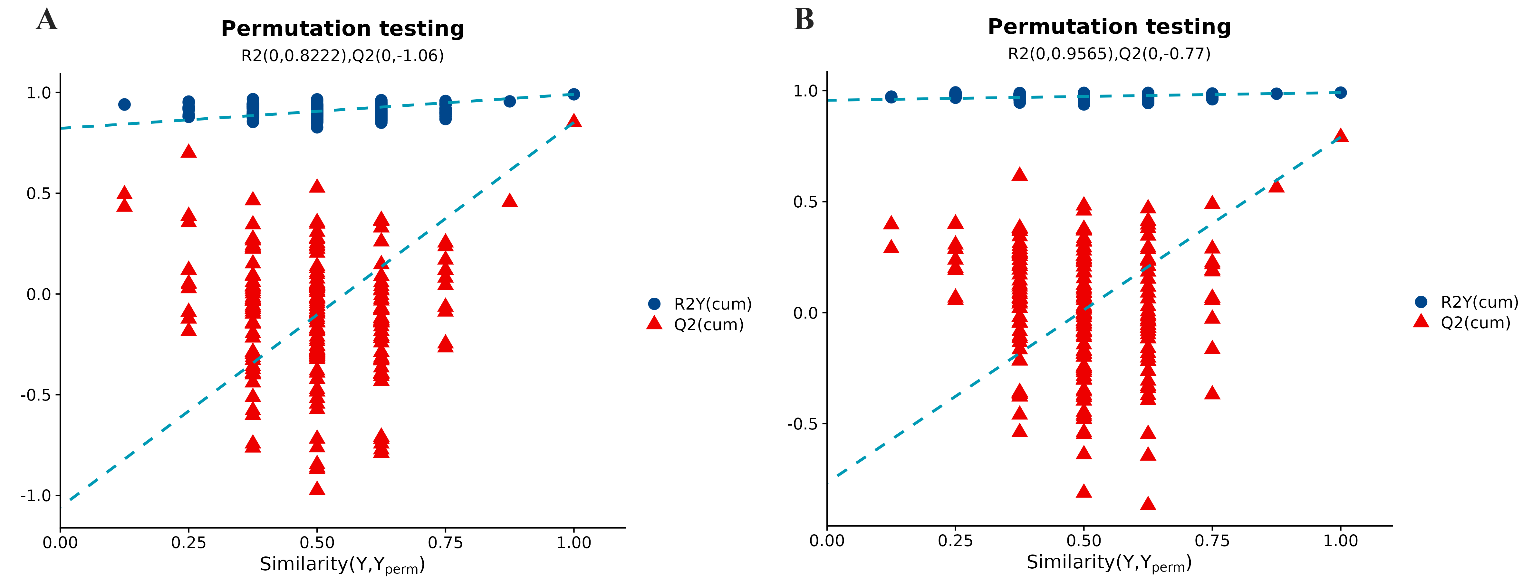


**Fig. S2.** Permutation test plot of OPLA-DA multivariate statistical analysis of liver metabonomics in db/db mice. **(A)** MD vs NC positive and negative ion patterns. **(B)** ATM-H vs MD positive and negative ion patterns.


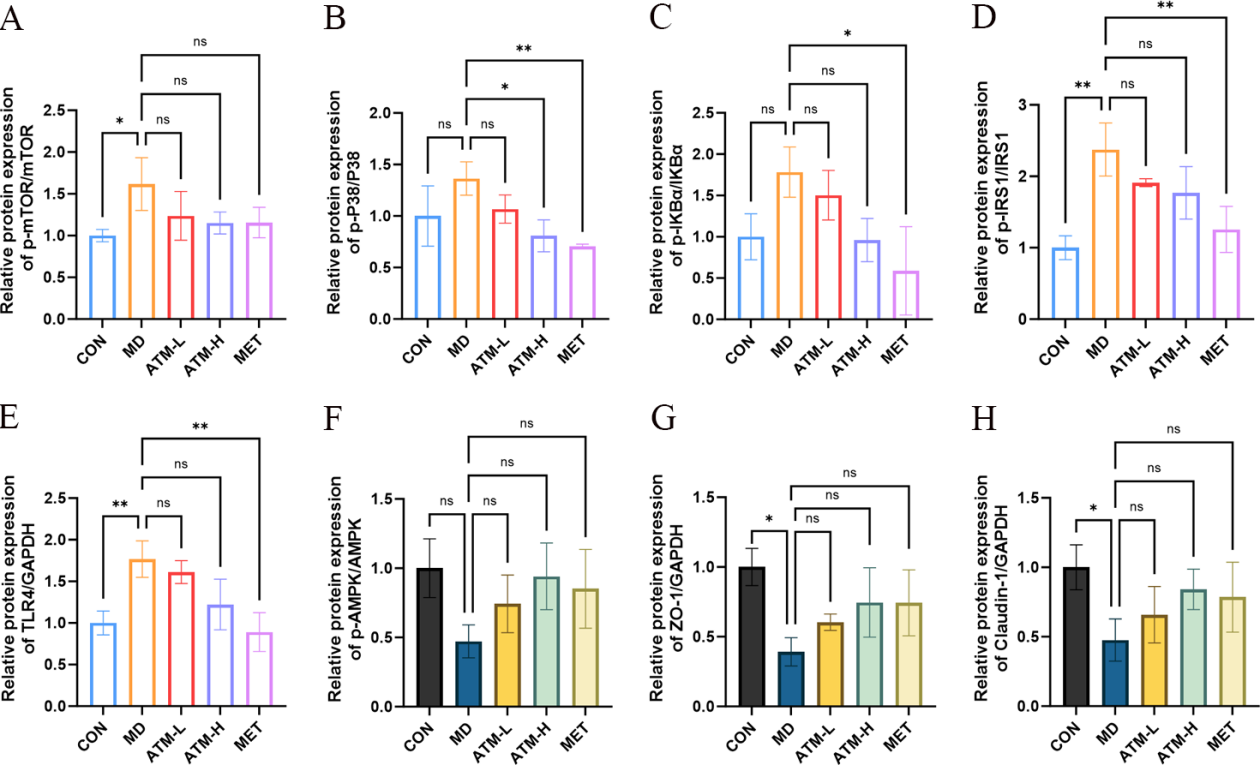


**Fig. S3.** Relative expression of AMPK/mTOR pathway-related proteins in liver **(A-E)** and colon **(F-H)**. *P < 0.05, ** P < 0.01.
